# Fused inverse-normal method for integrated differential expression analysis of RNA-seq data

**DOI:** 10.1186/s12859-022-04859-9

**Published:** 2022-08-05

**Authors:** Birbal Prasad, Xinzhong Li

**Affiliations:** National Horizons Centre, School of Health and Life Sciences, Teesside University, Darlington, DL1 1HG UK

**Keywords:** Meta-analysis, RNA-seq, Glioblastoma, Differential expression

## Abstract

**Background:**

Use of next-generation sequencing technologies to transcriptomics (RNA-seq) for gene expression profiling has found widespread application in studying different biological conditions including cancers. However, RNA-seq experiments are still small sample size experiments due to the cost. Recently, an increased focus has been on meta-analysis methods for integrated differential expression analysis for exploration of potential biomarkers. In this study, we propose a *p*-value combination method for meta-analysis of multiple independent but related RNA-seq studies that accounts for sample size of a study and direction of expression of genes in individual studies.

**Results:**

The proposed method generalizes the inverse-normal method without an increase in statistical or computational complexity and does not pre- or post-hoc filter genes that have conflicting direction of expression in different studies. Thus, the proposed method, as compared to the inverse-normal, has better potential for the discovery of differentially expressed genes (DEGs) with potentially conflicting differential signals from multiple studies related to disease. We demonstrated the use of the proposed method in detection of biologically relevant DEGs in glioblastoma (GBM), the most aggressive brain cancer. Our approach notably enabled the identification of over-expressed tumour suppressor gene *RAD51* in GBM compared to healthy controls, which has recently been shown to be a target for inhibition to enhance radiosensitivity of GBM cells during treatment. Pathway analysis identified multiple aberrant GBM related pathways as well as novel regulators such as *TCF7L2* and *MAPT* as important upstream regulators in GBM.

**Conclusions:**

The proposed meta-analysis method generalizes the existing inverse-normal method by providing a way to establish differential expression status for genes with conflicting direction of expression in individual RNA-seq studies. Hence, leading to further exploration of them as potential biomarkers for the disease.

**Supplementary Information:**

The online version contains supplementary material available at 10.1186/s12859-022-04859-9.

## Background

RNA sequencing (RNA-seq) technologies are now increasingly considered for whole transcriptome gene expression quantification studies as compared to traditional microarray technologies due to its high technical reproducibility and greater resolution [1]. Over the last decade, it has found widespread application in studying different biological conditions including cancers. For instance, sequencing data archived on The Cancer Genome Atlas (TCGA) (https://portal.gdc.cancer.gov/) have been used in several studies to explore potential biomarkers and mechanisms in oncogenesis [2, 3]. Despite its advantages and few large RNA-seq datasets [4, 5], RNA-seq experiments are still small sample size experiments because of its high cost. This leads to a problem of reduced statistical power in studies such as differential expression analysis where thousands of genes are studied at a time but only have tens to hundreds of samples. Combination of data or results from multiple independent but related studies (referred to as meta-analysis) have been widely used to increase available sample size and consequently the statistical power to obtain a precise estimate of gene expression differentials [6, 7]. In the context of differential expression analysis, several different meta-analysis approaches have been proposed for integrating microarray studies [8, 9] and some of them have later been adapted for RNA-seq data [10, 11].

For microarray gene expression studies, apart from vote-counting and direct merging of datasets, meta-analysis methods can mainly be classified into three types based on the combined statistic [7]. First are methods based on effect-size combination in which a combined effect (for instance, strength of differential expression between two conditions for a gene) is obtained based on the calculated effect sizes and its variance. Two possible models namely, fixed and random effects model are used to obtain the combined effect [12]. Second are approaches based on integration of *p*-values obtained from per-study analysis into a single combined *p*-value per gene [13]. Lastly, are approaches based on rank combination which are non-parametric and allow for integration of studies based on a statistic that can be ordered, e.g., fold change of a gene [14]. However, RNA-seq data are counts data, i.e., normalized number of sequenced reads within a certain gene or transcript, unlike the microarray data which are continuous, e.g., normalized signal intensity of image [15]. Hence, the methods initially proposed for microarray data are not suited to be applied directly to RNA-seq data in many cases [10].

In case of RNA-seq data, Poisson or Negative-Binomial distributions are typically used to model gene counts [16]. Kulinskaya et al. [17] described an effect-size combination method using an Anscombe transformation of Poisson distributed data. However, as highlighted by Rau et al. [10], this effect-size combination approach is not appropriate for RNA-seq data due to over-dispersion among biological replicates and presence of zero-inflation. Rau et al. [10] considered two *p*-value combination methods, namely Fisher and inverse normal (IN) or Stouffer’s methods, previously proposed and used for meta-analysis of microarray studies [8, 9, 13] and demonstrated how these can be adapted in RNA-seq data analysis. Their results illustrated that Fisher and IN methods were very similar to each other in terms of performance but were better than the global and per-study differential analysis [10]. These two (Fisher and IN) *p*-value combination approaches have been implemented in several R packages, e.g., metaRNASeq [10], metaseqR [18] and metaSeq [19] and are the most widely used methods due to its statistical simplicity and ease of direct application for meta-analysis of RNA-seq studies for differential expression.

Among all the existing meta-analysis methods for RNA-seq data discussed above, only few of the *p*-value combination methods (e.g., IN and PANDORA *p*-value [18]) allow for incorporation of information regarding the number of replicates in different studies to be combined through specification of a set of weights. However, information related to the direction of expression (up- or down-regulated) of a gene across different studies is not accounted for or included in any of these meta-analysis methods for RNA-seq data. Under- and over-expressed genes are analysed together and genes exhibiting conflicting direction of expression across studies are either removed prior to meta-analysis or are suggested to be identified and removed post-hoc [10, 11]. Hence, no conclusion can be drawn with regards to differential expression for the genes that have conflicting direction of expression across different studies. Given that a significant proportion of genes may exhibit conflicting direction of expression across different gene expression studies [20], particularly when more and more RNA-seq data are publicly available and included into integration, emphasis is warranted on including this important prior information in a meta-analysis setting.

Recently, importance of inclusion of direction of expression information for genes in RNA-seq meta-analysis has been recognized leading to a generalization of some existing *p*-value combination methods such as Fisher method and Bayesian Hierarchical Model [21–23]. However, these generalizations come at a cost of increased statistical and computational complexity which discourages their widespread application to transcriptomic studies. In this study, we aimed to develop a new approach for integrated differential meta-analysis of RNA-seq data which accounts for both the sample size and direction of gene regulation in each study. The proposed approach leads to a generalization of the IN method without introducing additional statistical or computational cost and hence is simple and intuitive for real data application. First, we propose a modified inverse-normal (MIN) approach for *p*-value combination and assess its performance by comparing it with the IN method based on an extensive simulation study. Next, to overcome the limitation of MIN method, we further propose a fused inverse normal (FIN) method for *p*-value combination and assess its performance by comparing it to IN and MIN methods in a simulation study. Then an application to a set of real glioblastoma (GBM, the most aggressive type of brain cancer) studies has been conducted. Moreover, we assessed the relevance of the identified differentially expressed genes (DEGs) by FIN method for GBM by using Ingenuity Pathway Analysis (IPA, www.qiagen.com/ingenuity) for pathway analysis and upstream regulator analysis (URA).

## Methods

Let ${y}_{gcrs}$ be the observed count for gene $g$ ($g=1, 2, \dots , G$) in condition $c$ ($c=1, 2$) of biological replicate $r$ ($r=1, 2, \dots , {R}_{cs})$ in study $s$ ($s=1, 2, \dots , S$). For an integrated differential analysis of gene expression across multiple studies, we first conducted the differential expression analysis within a given study $s$ using edgeR package (version 3.26.5) in R version 3.6.0 [24] with likelihood ratio test as the test for differential expression. Let ${p}_{gs}$ be the raw *p*-value for per-gene and per-study obtained using the individual differential expression analysis within a given study $s$ for gene $g$. The null hypothesis tested in the individual differential analysis is that the gene is non-differentially expressed in the particular study. For notational convenience, the notations similar to the ones used in Rau et al. (2014) [10] were adopted in this study.

### Modified inverse-normal method

Let ${B}_{gs}$ be a Bernoulli random variable which takes values 1 and -1 when a gene $g$ is over- and under-expressed respectively in a study $s$. A gene can be assessed as over- or under-expressed based on the fold change values (> 1 or < 1) of the gene in a study. Then, for a gene $g$, we define a combined statistic

1 $${N}_{g}=\sum_{s=1}^{S}{w}_{s}{B}_{gs}|{\Phi }^{-1}\left(1-{p}_{gs}\right)|$$

where ${w}_{s}$ are a set of study specific weights described by Marot and Mayer [25] as follows:

2 $${w}_{s}=\sqrt{\frac{\sum_{c}{R}_{cs}}{{\sum }_{k}{\sum }_{c}{R}_{ck}}}$$

Here, ${\sum }_{c}{R}_{cs}$ is the total number of biological replicates in a study $s$ for all condition *c* and ${\sum }_{k}{\sum }_{c}{R}_{ck}$ indicates the total number of biological replicates in all studies. Moreover, ${N}_{g}$ can be considered as a weighted z-score. An advantage of this weighting criteria is that larger weights are attributed to studies with larger sample sizes. $\Phi$ is the standard normal cumulative distribution function and ${p}_{gs}$ is the raw *p*-value obtained for gene $g$ by differential analysis for study $s$.

It is assumed that ${p}_{gs}$ are uniformly distributed under the null hypothesis (${H}_{0}$) leading to ${\Phi }^{-1}(1-{p}_{gs})$ being standard normal in the above formula (1). However, this assumption of ${p}_{gs}$ is not automatically satisfied when dealing with RNA-seq data [10]. Filtering of very low expressed genes in each study results in *p*-values which are roughly uniformly distributed under the null hypothesis [10]. Similarly, under ${\mathrm{H}}_{0}$, ${\mathrm{B}}_{\mathrm{gs}}$ is a Bernoulli random variable taking values 1 and -1 with equal probability. This is because under ${\mathrm{H}}_{0}$, a gene is non-differentially expressed. Hence, the chance of it being over- or under-expressed in a study is the same. However, note that since in a particular study we have both differentially and non-differentially expressed genes, the numbers of over-expressed genes in c1 and c2 are not expected to be the same. Then, we have that ${B}_{gs}\left|{\Phi }^{-1}\left(1-{p}_{gs}\right)\right| \sim N(0, 1)$ (see Theorem 1).

#### Theorem 1

Let $X$ and $Y$ be two independent random variables where $X\sim N(0, 1)$ and $Y$ is a Bernoulli random variable taking values $1$ and $-1$ with equal probability. Then, $Z=Y|X|$ is standard normal distributed.

#### *Proof*

Using first principle,

3 $${\mathbb{P}}[Y\left|X\right|\le t]={\mathbb{P}}\left[Y=1, \left|X\right|\le t\right]+{\mathbb{P}}\left[Y=-1, -\left|X\right|\le t\right]=\frac{1}{2}{\mathbb{P}}\left[\left|X\right|\le t\right]+\frac{1}{2}{\mathbb{P}}\left[\left|X\right|\ge -t\right]$$

Now, if $t<0$, the RHS of $(3)$ becomes $\frac{1}{2}{\mathbb{P}}[\left|X\right|\ge -t]$. By symmetry of the normal distribution, we have

$${\mathbb{P}}\left[Y\left|X\right|\le t\right]={\mathbb{P}}\left[X\le t\right]=\Phi (t)$$

where $\Phi$ is the cumulative distribution function of standard normal.

For $t\ge 0$, the RHS of $(3)$ becomes $\frac{1}{2}{\mathbb{P}}\left[\left|X\right|\le t\right]+\frac{1}{2}$. Hence, by symmetry of the normal distribution, we have

$${\mathbb{P}}\left[Y\left|X\right|\le t\right]={\mathbb{P}}\left[X\in \left[0, t\right]\right]+\frac{1}{2}={\mathbb{P}}\left[X\le t\right]=\Phi (t)$$

Thus, $$Z\sim N(0, 1)$$. ■

Hence, ${N}_{g}$ in Eq. (1) is a linear combination of independent standard normal variables. Thus, is also standard normal. A two-sided test can then be performed with ${H}_{0}$ being that the gene $g$ is not differentially expressed between two conditions (case vs control) and combined *p*-value is given by (${p}_{g}={\mathbb{P}}\left(\left|z\right|\ge {N}_{g}\right)$, i.e.

$${p}_{g}=2[1-\Phi \left({|N}_{g}|\right)]$$

A correction for multiple testing to control the false discovery rate (FDR) at a desired level $\alpha$ can be done by Benjamini–Hochberg (BH) approach [26].

### Fused inverse-normal method

To address the conservative nature of MIN method (see simulation study results), we propose a mixture method which is a mixture of IN and MIN method for integrated differential analysis. In contrast to formula (1) we define ${N}_{g}$ as follows:

4 $${N}_{g}=\left\{\begin{array}{l}\sum_{s=1}^{S}{w}_{s}{\Phi }^{-1}\left(1-{p}_{gs}\right), if \,g \,has \, same \, direction \, of\, expression\, across\, s\\ \sum_{s=1}^{S}{w}_{s}{B}_{gs}\left|{\Phi }^{-1}\left(1-{p}_{gs}\right)\right|, otherwise\end{array}\right.$$

Here, ${w}_{s}$, $\Phi$ and ${B}_{gs}$ have their usual meaning as described previously. As ${N}_{g}$ follows a standard normal distribution given the assumption that ${p}_{gs}$ is uniformly distributed under the null hypothesis, a one-sided test on the right-hand tail of the distribution (as proposed in [10]) can be performed for genes with same direction of expression across studies. For the genes with conflicting direction of expression across studies, a two-sided test can be performed. ${H}_{0}$ being the same in both the cases. Multiple testing correction to control the overall FDR can then be carried out using the BH method. A detailed interpretation of the FIN method in terms of differential expression of a gene and its direction of expression in individual studies can be found in Additional file 1: Supplementary 1 (Interpretations of the FIN method section).

### Simulation study

To investigate the performance and compare the MIN and FIN methods with state-of-the-art *p*-value combination method (IN with post-hoc filtering), we performed a simulation study. The simulation study has been divided into two parts. In first part of the simulation study, we compare MIN with IN to understand the behaviour of MIN in comparison to IN and emphasize the need for FIN method. Next, we assess and compare the performance of FIN method to that of IN and MIN methods.

An extensive set of RNA-seq data was generated using the negative binomial distribution for the counts ${y}_{gcrs}$ and method described in Rau et al. [10] (see Additional file 1: Supplementary 1, section: Simulation study model). Parameters for the simulation study were estimated from a real RNA-seq dataset for Alzheimer’s disease (AD) study downloaded from Gene Expression Omnibus (GEO, https://www.ncbi.nlm.nih.gov/geo/) [27] with accession number GSE125583 which contains data for 219 AD patients and 70 normal control subjects. The method used for estimation of mean and dispersion parameters from GSE125583 were as described in Rau et al. [10] with BH *p*-value < 0.05 being used to classify a gene as a DEG. This dataset was chosen as it has considerable number of samples for both biological conditions, namely case and control. Simulation settings for inter-study variability parameter ($\sigma )$, number of samples per condition and number of studies have been detailed in Table 1. The inter-study variability parameter represents the amount of variability between the studies considered for meta-analysis. In practice, the observed variability between human data studies is considerable ($\sigma \sim 0.5$) [10]. We chose two different values of $\sigma$ (0.15 and 0.5) to represent small and large amount of inter-study variability respectively. For each setting described in Table 1, 100 independent trials were considered.

**Table 1:** Simulation settings for inter-study variability parameter ($\sigma$), number of studies and number of replicates per study

Setting	$\sigma$	No. of studies	No. of replicates (case, control)	AUC (MIN, IN, FIN)	Std. dev (MIN, IN, FIN)
1	0.15	3	(10, 10) (15, 10) (12, 16)	0.886, 0.920, 0.920	0.005, 0.003, 0.003
2	0.15	5	(10, 10) (15, 10) (12, 16) (14, 12) (20, 20)	0.953, 0.970, 0.970	0.005, < 0.001, 0.001
3	0.5	3	(10, 10) (15, 10) (12, 16)	0.950, 0.965, 0.966	0.004, 0.005, 0.005
4	0.5	5	(10, 10) (15, 10) (12, 16) (14, 12) (20, 20)	0.957, 0.977, 0.977	0.005, 0.005, 0.005

Area under the receiver operating characteristic curves (AUC) for inverse-normal (IN), modified inverse-normal (MIN) and fused inverse-normal (FIN) methods computed using 100 trials for each simulation setting. Std. dev: Standard deviation

For each simulation setting, individual *p*-values obtained from differential expression analysis using edgeR (version 3.26.5) were combined using MIN, FIN and IN methods. A gene was considered differentially expressed if the BH adjusted combined *p*-value (FDR) $<0.05$. Next, based on area under the receiver operating characteristic (ROC) curves (AUC), the meta-analysis methods were assessed for detection power in identifying DEGs under all simulation settings. Furthermore, the characteristics of MIN and FIN methods were also assessed in terms of: (a) FDR, (b) the proportion of true-positives (TPs) among the unique DEGs identified by each of the two methods as compared to IN method and (c) proportion of truly unique DEGs with the observed effective direction of expression as the true direction of expression.

### Application to brain cancer data

To demonstrate how the MIN and FIN method can be adapted in practice for differential meta-analysis of RNA-seq data and compare it with IN method, an application to real glioblastoma (GBM) studies has been conducted.

#### Data collection and pre-processing

GBM RNA-seq datasets were searched in GEO (https://www.ncbi.nlm.nih.gov/geo/) and TCGA databases (https://portal.gdc.cancer.gov/). Datasets were selected based on a selection criterion that at least 3 GBM patients and 3 normal tissue samples are available for analysis. Three different GBM RNA-seq datasets, two from GEO (with accession ID: GSE123892 and GSE151352) and one from TCGA (TCGA-GBM) matched our selection criteria and were considered for analysis (for details, see Table 2). Raw gene or transcript counts data (where available) was directly downloaded for TCGA-GBM and GSE123892 datasets. For GSE151352, raw FASTQ files were downloaded and processed using Galaxy web platform via the European UseGalaxy server (https://usegalaxy.eu/) [28] to obtain raw counts. The quality of the raw reads was assessed (using FastQC) and the specified adapter sequence ATCACCGACTGCCCATAGAGAGGCTGAGAC was removed with Cutadapt (version 1.16) [29]. The parameters used for this step were the parameters provided by the submitter of the dataset on GEO. The adapter trimmed reads were aligned to the reference genome (GRCh37.p13) using sequence aligner RNA STAR (Galaxy version 2.7.5b) [30] where other parameters used were default settings. Following alignment, the generated BAM files were processed using the featureCounts tool (Galaxy version 1.6.4 + galaxy2) [31] to get raw counts for each RNA-seq data sample. More details of the processing pipeline used for GSE151352 can be found in Additional file 1: Figure S1 and processing of raw RNA-seq dataset GSE151352 using GALAXY section.

**Table 2:** Information about GBM RNA-seq datasets used for integrated analysis using different *p*-value combination methods in our study

Datasets	No. of replicates (cases/normal)	No. of genes (after filtering)	Up DEGs	Down DEGs
GSE123892	4/3	15,024	1914	1837
GSE151352	12/12	12,916	670	1545
TCGA-GBM	160/5	17,943	3746	3183

Up and down differentially expressed genes (DEGs) refer to the up and down-regulated DEGs obtained in per-study differential analysis

#### Per-study differential expression analysis

The raw counts of each of these datasets (TCGA-GBM, GSE123892 and GSE151352) were processed separately for quality control and differential expression analysis using edgeR package (version 3.26.5) in R. Raw counts data (transcript) were annotated by mapping Ensembl IDs to Entrez Gene IDs and gene symbols (org.Hs.eg.db package, version 3.8.2 in R [32]). Ensembl IDs with no Entrez ID mapping were filtered out. For those with multiple matchings, the one with highest aggregated count was selected. Counts per million (CPM) threshold (0.85 CPM) was carefully selected to reduce the number of low expressed transcripts [33]. Although subjective, this choice of threshold seems to work well for the uniform distribution assumption for the *p*-values under ${H}_{0}$. Only genes left after low expression filtering were considered for individual differential expression analysis in order to satisfy the uniformity assumption on *p*-values under ${H}_{0}$. The remaining transcripts were then normalized using the trimmed mean of M-values (TMM) method [34]. Common and tag-wise dispersion were estimated and a negative-binomial generalized log-linear model was fitted to the read counts using the glmFit function under the edgeR package. Raw *p*-values were then obtained from the differential analysis for case/control conditions.

#### Meta-analysis

Once the raw *p*-values were obtained from the individual differential expression analysis for each dataset, IN, MIN and FIN methods were applied for *p*-value combination. Since the TCGA-GBM dataset (160 GBM vs 5 normal samples) is much larger in terms of number of samples as compared to GSE123892 (4 GBM vs 3 normal samples) and GSE151352 (12 GBM vs 12 normal samples), we considered two different combination scenarios. First, all TCGA-GBM samples were used for individual analysis to obtain the raw *p*-values. Second, 20 cases and 5 normal samples randomly selected from TCGA-GBM dataset were considered for individual analysis to get raw *p*-values and then considered for meta-analysis with the other two datasets (GSE123892 and GSE151352). 10 different random selections were made, and individual differential expression analysis were conducted respectively. Second scenario ensured that the datasets included in meta-analysis had comparable sample sizes.

For each of the combination methods, we assessed the number of DEGs based on average absolute log fold-change $\sum_{i=1}^{n}\left|{\mathrm{log}}_{2}F{C}_{i}\right|/n>1$ and FDR *p*-value $<0.05$ criteria. Here, $n$ denotes the number of datasets in which a particular gene was present. In case a gene was absent in a dataset, the weights in the combination methods were estimated only using the number of replicates in datasets in which the gene was present. The three *p*-value combination methods were then compared based on number of DEGs identified and unique DEGs identified by each method.

#### Pathway analysis and biological significance

DEGs obtained by the FIN method were further explored to assess their biological relevance to GBM. QIAGEN’s Ingenuity Pathway Analysis (IPA) (www.qiagen.com/ingenuity) tool was used to identify biological pathways in which DEGs were enriched and upstream regulator analysis (URA) identified upstream regulators for GBM. We performed pathway analysis and URA separately for DEGs that were up-regulated and down-regulated and were present in all three datasets.

## Results

Both MIN and FIN methods were compared to the IN (with post-hoc filtering) method in a simulation study and a real data application.

### Simulation study

#### MIN and IN comparison

**Fig. 1:**
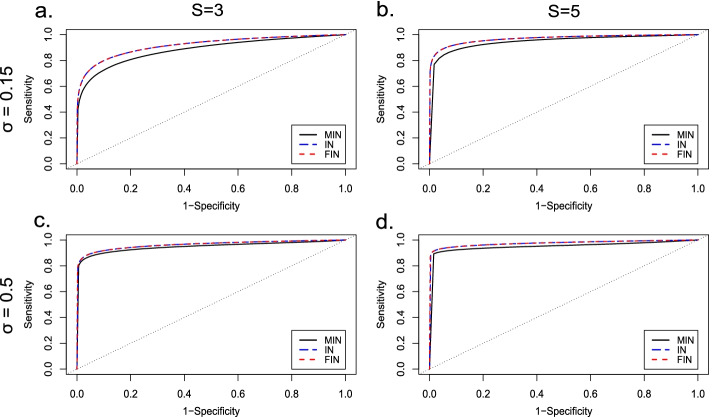
Performance comparison of modified inverse-normal, inverse-normal and fused inverse-normal methods. Plots of receiver operating characteristics (ROC) curves averaged over 100 trials for each simulation setting for all three methods. Simulation settings are represented by rows (from top to bottom): corresponding to low (σ = 0.15) and high (σ = 0.5) inter-study variability and columns (from left to right): corresponding to 3 (S = 3) and 5 studies (S = 5) combined. The black, blue, and red ROC curves represent the modified inverse-normal (MIN), inverse-normal (IN) and fused inverse-normal (FIN) methods respectively

Based on AUC (Table 1, Fig. 1), both meta-analysis methods (MIN and IN) performed well in terms of detection power in identifying DEGs (BH adjusted combined *p*-value $<0.05$) under all simulation settings. For both low ($\sigma =0.15$, Fig. 1a-b) and high ($\sigma =0.5$, Fig. 1c–d) inter-study variability, we observed that the MIN method was more conservative for true DEGs (AUC was smaller) than the IN method. However, as the inter-study variability and the number of studies to be combined increased, both meta-analysis methods were found to have comparable performance (Fig. 1c–d). Although slightly conservative in its performance with respect to the IN approach, MIN method has the advantage of using direction of expression information leading to identification of DEGs among genes with conflicting direction of expression across studies. The conservative behaviour of the MIN method can be attributed to the fact that a two-sided hypothesis testing is performed as compared to a one-sided test on right-hand tail of the distribution in case of IN method. Hence, next we proposed the FIN method as a mixture of IN and MIN methods to circumvent the issue of conservativeness of MIN method among true DEGs.

**Fig. 2:**
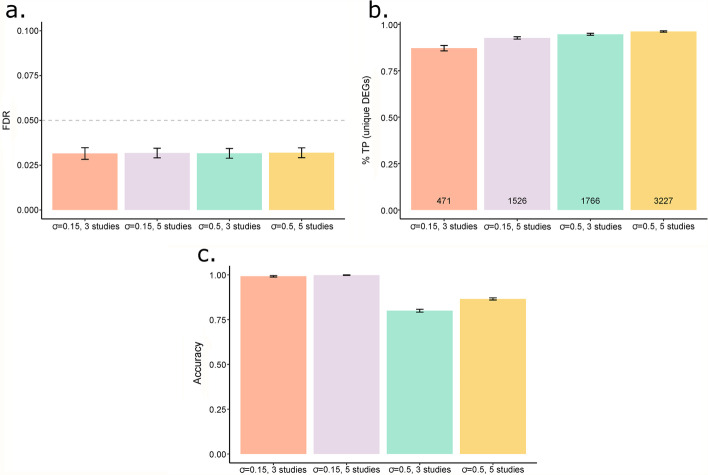
Characteristics of modified inverse-normal method. **a** False discovery rates (FDR) for modified inverse-normal (MIN) method for all simulation settings. **b** Proportion of true positives (TPs) among unique differentially expressed genes (DEGs) identified by MIN method as compared to inverse-normal (IN) method. **c** Proportion of truly unique DEGs (MIN) with the observed effective direction of expression as the true direction of expression

As expected, with increase in inter-study variability and number of studies to be combined, the number of genes with mismatched direction of expression was significantly higher (see Additional file 1: Table S1). We also note that the FDR for all simulation settings was controlled well below 5% threshold (Fig. 2a). In terms of uniquely identified DEGs by the MIN method as compared to IN method, the proportion of true positives (TPs) was higher than 80% (Fig. 2b) in all simulation settings. A large proportion of TPs among the unique DEGs identified by the MIN method indicates that the MIN approach can lead to DEGs that are biologically relevant to a disease in a real application. Moreover, as the inter-study variability, number of studies or both increased, there was an increase in the number of uniquely identified DEGs by the MIN method and proportion of TPs among them (Fig. 2b). More importantly, a high percentage of these truly unique DEGs (~ 80% or more in all settings) were observed to have the true direction of expression (Fig. 2c) suggesting that a significantly high percentage of uniquely identified DEGs by the MIN method in real data applications will have true direction of expression as their effective direction of expression. The effective observed direction of expression was determined by the sign of ${N}_{g}$ as defined in Eq. (1).

#### FIN, IN and MIN comparison

**Fig. 3:**
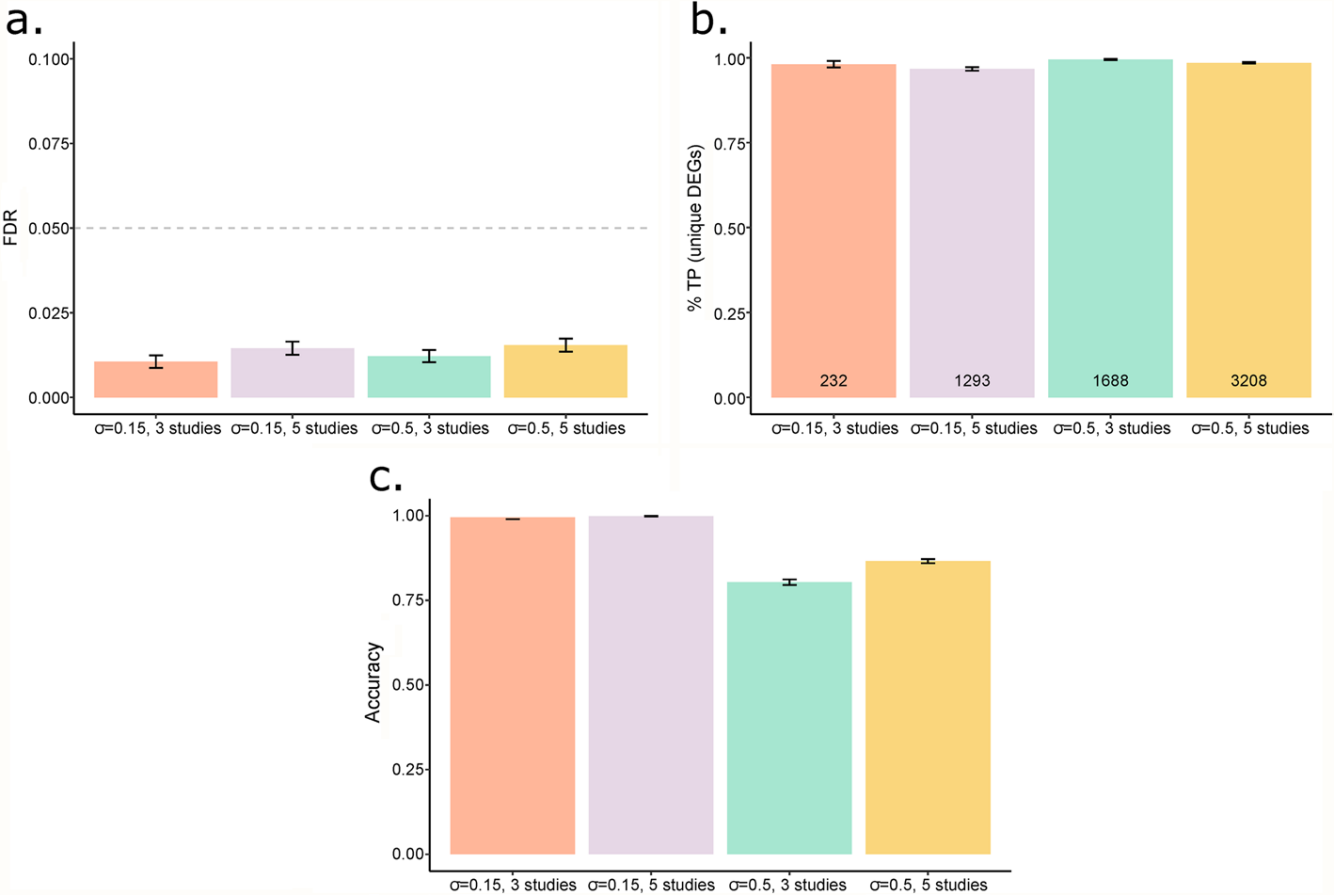
Characteristics of fused inverse-normal method. **a** False discovery rates (FDR) for fused inverse-normal (FIN) method for all simulation settings. **b** Proportion of true-positives (TPs) among unique differentially expressed genes (DEGs) identified by FIN method as compared to inverse-normal method. **c** Proportion of truly unique DEGs (FIN) with the observed effective direction of expression as the true direction of expression

In addition to the simulation study for comparing MIN with IN method, we assess and compare the performance of FIN method to that of IN and MIN methods by using the same simulated data and settings described in Table 1. Based on AUC (Table 1, Fig. 1), FIN performed similar or better than IN method and had better performance than MIN under all simulation settings. As with MIN, FIN method also has the advantage of using direction of expression information and hence identified DEGs among genes with conflicting direction of expression in contrast with IN method. More importantly, we observed that FIN significantly improved detection power for true DE genes with concordant differential expression patterns across studies as compared to MIN method and does not lead to increased number of false positive detections overall (Fig. 3a).

As compared to IN, the proportion of TPs among the uniquely identified DEGs by FIN method was higher than 90% (Fig. 3b) indicating that FIN method can lead to DEGs that are biologically relevant to a disease in a real application. Similar to MIN, as the inter-study variability, number of studies or both increased, there was an increase in the number of uniquely identified DEGs by the FIN method as compared to IN method and proportion of TPs among them (Fig. 3b). In addition, a high percentage of these truly unique DEGs (> 80% in all settings) were observed to have the true direction of expression (Fig. 3c) suggesting that a significantly high percentage of uniquely identified DEGs by the FIN method in real data applications will have true direction of expression as their effective direction of expression. The effective observed direction of expression was determined by the sign of ${N}_{g}$ for genes with conflicting direction of expression across studies. In case of same direction of expression of a gene across studies, the consistent direction of expression was kept as the effective direction of expression.

### Glioblastoma brain cancer data application

#### Per-study differential expression analysis

DEGs were identified in per-study differential analysis based on the criteria $\left|{\mathrm{log}}_{2}FC\right|>1$ and FDR *p*-value $<0.05$ and can be found in Table 2. We note that TCGA-GBM dataset has a much larger library size (~ 47 million reads, Illumina HiSeq 2000 v2 sequencer) as compared to GSE151352 (~ 4 million reads, Ion Torrent S5 sequencer) and GSE123892 (~ 35 million reads, Illumina HiSeq 2500 sequencer). Hence, we observed a differing number of genes left after filtering and consequently a much larger number of DEGs being observed for TCGA-GBM dataset as compared to the other two (Table 2) in per-study differential expression analysis. As the sequencing output gets larger, the smaller count differences between samples are declared significant by models for differential expression in edgeR. A more detailed treatment of differential expression in RNA-seq data and how it is affected by sequencing depth and other factors can be found in Tarazona et al. [35].

Moreover, we also considered individual differential analysis for TCGA-GBM RNA-seq data by randomly selecting 20 cases together with available 5 normal samples in order to make all three datasets (GSE123892, GSE151352 and TCGA-GBM) comparable in terms of number of replicates for the meta-analysis (see Additional file 1: Table S2). Hence, we considered two different meta-analysis scenarios.

#### Meta-analysis

**Fig. 4:**
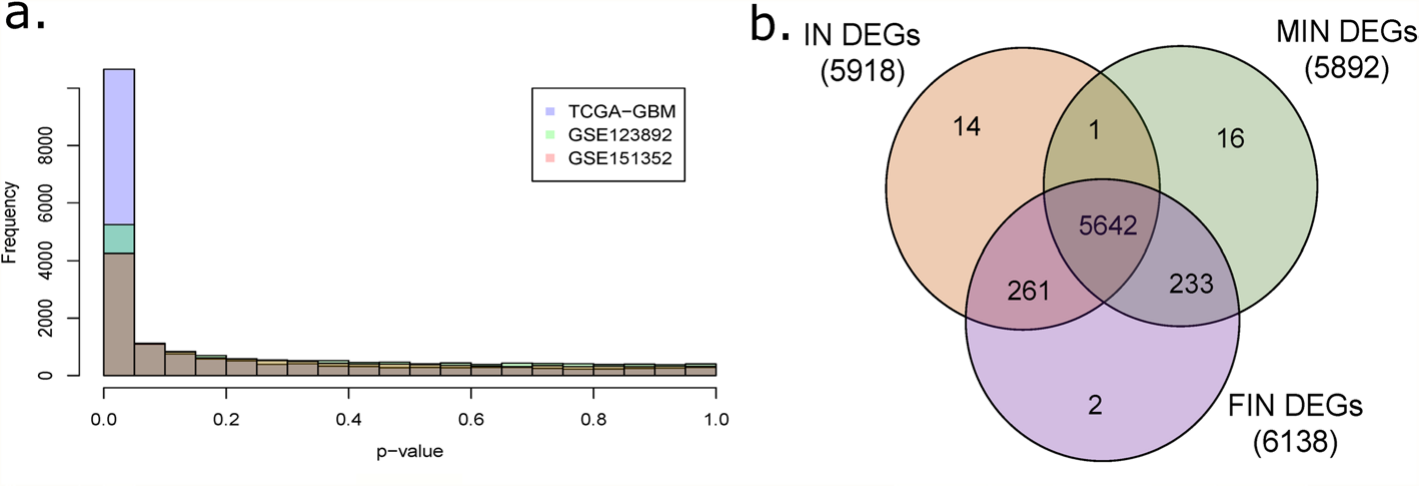
Comparison of results from meta-analysis methods. **a** Histograms of raw *p*-values obtained from per-study differential analysis of GSE123892 and GSE151352 and TCGA-GBM datasets used in real data application. **b** Venn diagram of the differentially expressed genes (DEGs) identified using inverse-normal (IN), modified inverse-normal (MIN) and fused inverse-normal (FIN) methods

In scenario one (GSE123892, GSE151352 and TCGA-GBM with all 165 samples), a total of 18,315 unique gene pool was considered for meta-analysis which was the combination of genes identified in each RNA-seq data analysis after quality control and filtering (Table 2). 13,056 out of 18,315 genes (~ 71%) were found to have the same direction of expression across the studies in which they were present whereas 5259 (~ 29%) of genes had conflicting or mismatched direction of expression. The direction of expression for a gene in an individual study was determined based on the sign of ${\mathrm{log}}_{2}FC$ obtained for that gene in per-study differential analysis. Hence, only 13,056 genes were effectively considered for IN method as compared to 18,315 genes for MIN and FIN methods for identifying DEGs because of post-hoc removal of DEGs with conflicting direction of expression in the IN method. Importantly, the uniform distribution assumption under the null hypothesis for the raw *p*-values of the considered gene pool was found to be appropriate (Fig. 4a).

A total of 5918, 5892 and 6138 DEGs were identified by the IN, MIN and FIN methods respectively. Of the DEGs detected by all these meta-analysis methods, more than 90% of them were in common (Fig. 4b) with FIN method having a higher detection power than the other two methods. 261 DEGs were found to be common between the IN and FIN methods but were not identified by the MIN method. This subset of 261 DEGs was characterized by low values of the combination statistic ${N}_{g}$ (largest value of 2.22) and consistent direction of expression across studies. In addition, a large number among them (177 out of 261 DEGs, ~ 68%) were present in just one of the three studies. 50 and 34 out of 261 DEGs were present in two and all three considered studies respectively.

MIN and FIN methods identified a total of 233 and 235 DEGs with mismatched direction of expression across studies by incorporating the direction of expression information. All 233 DEGs with mismatched direction of expression across studies identified by MIN were also identified by the FIN method. More importantly, in the subset of DEGs which were present in all three datasets, 5.26% of DEGs had conflicting direction of expression across studies. Although, small in proportion, this would be of importance in case a gene of interest for the disease being studied has conflicting direction of expression across different studies. Particularly when more datasets are included in meta-analysis, the number of genes considered in IN approach can be massively reduced.

Given that the FIN method has the highest power of DEG detection, we further explore the DEGs obtained using this meta-analysis procedure for biological significance. Top 10 up and down-regulated DEGs identified by FIN method are presented in Table 3. For full list of DEGs identified by different meta-analysis methods, see Additional files 2, 3, 4: Supplementary S2, S3 and S4. In terms of effective direction of expression of DEGs, 2914 DEGs with same direction of expression across studies and 180 DEGs with mismatched direction of expression were up-regulated. Similarly, 2989 (same direction) and 55 (mismatched direction) DEGs were down-regulated.

**Table 3:** Top 10 up- and down-regulated differentially expressed genes (DEGs) identified by the fused inverse-normal method

DEGs (Up)	${N}_{g}$	Mean |logFC|	Effect	BH *p*-value	DEGs (Down)	${N}_{g}$	Mean |logFC|	Effect	BH *p*-value
*EIF4EBP1*	10.45	3.33	+++	$<1.62\times {10}^{-15}$	*SMAD12*	11.19	4.32	−−−	$<1.62\times {10}^{-15}$
*WEE1*	10.39	4.04	+++	$<1.62\times {10}^{-15}$	*RASGRF2*	11.10	4.19	−−−	$<1.62\times {10}^{-15}$
*VIM*	10.39	3.68	+++	$<1.62\times {10}^{-15}$	*DNAJC6*	11.07	3.71	−−−	$<1.62\times {10}^{-15}$
*NUSAP1*	10.29	4.67	+++	$<1.62\times {10}^{-15}$	*SERPINI1*	10.99	4.79	−−−	$<1.62\times {10}^{-15}$
*HJURP*	10.24	5.79	+++	$<1.62\times {10}^{-15}$	*ATP1B1*	10.98	3.35	−−−	$<1.62\times {10}^{-15}$
*KIF4A*	10.15	4.48	+++	$<1.62\times {10}^{-15}$	*ATP8A1*	10.91	3.95	−−−	$<1.62\times {10}^{-15}$
*KIF20A*	10.12	5.80	+++	$<1.62\times {10}^{-15}$	*JAKMIP3*	10.91	4.40	−−−	$<1.62\times {10}^{-15}$
*AURKB*	10.09	5.48	+++	$<1.62\times {10}^{-15}$	*MFSD6*	10.90	2.83	−−−	$<1.62\times {10}^{-15}$
*UBE2C*	10.07	5.95	+++	$<1.62\times {10}^{-15}$	*DCTN1-AS1*	10.88	5.33	−−−	$<1.62\times {10}^{-15}$
*CCNB2*	10.04	4.63	+++	$<1.62\times {10}^{-15}$	*PRKACB*	10.85	2.35	−−−	$<1.62\times {10}^{-15}$

The DEGs have been sorted based on the value of the statistic ${N}_{g}$ and the mean of absolute value of the ${\mathrm{log}}_{2}FC$ have been reported. Effect signifies the direction of expression of DEGs in the per-study differential analysis. BH *p*-value: Benjamini Hochberg *p*-value

In scenario 2 (GSE123892, GSE151352 and TCGA-GBM with 10 random selections of 20 cases and 5 normal samples), the identified DEGs were consistent with scenario 1. For instance, on average using the FIN method, about 94% of the DEGs obtained when randomly selected subset was considered were also found in DEGs identified using the full TCGA-GBM dataset (see Additional file 1: Table S3). Hence, suggesting that the identification of DEGs was stable across these two settings. Results for scenario 2 for random selection have been detailed in Additional file 1: Table S4, S5 and S6.

#### Pathway analysis and biological significance

We performed pathway analysis and URA separately for FIN method DEGs that were up-regulated and down-regulated and were present in all three datasets. We also note that not all identified DEGs by the meta-analysis methods are present in all three studies considered. Number of DEGs present in one, two or all three datasets have been detailed in Table 4.

**Table 4:** Number of differentially expressed genes (DEGs) found in one, two or all three datasets

Method	Expression direction	Present in one study	Present in two studies	Present in three studies	Total DEGs
IN	Same Mismatched	1368 0	1085 0	3465 0	5918
MIN	Same Mismatched	1182 0	1035 52	3442 181	5892
FIN	Same Mismatched	1359 0	1083 53	3461 182	6138

Same and mismatched represents if the direction of expression of a DEG was consistent across a study or not respectively. IN: Inverse-normal, MIN: Modified inverse-normal, FIN: Fused inverse-normal

Of 1798 up-regulated DEGs, all of them mapped in the IPA database and 101 canonical pathways were identified based on BH adjusted *p*-value (< 0.01). These include Heptatic Fibrosis Signaling Pathway (adj. Pval. $=3.98\times {10}^{-13}$, ratio = 0.205), Kinetochore Metaphase Signaling Pathway (adj. Pval. $=7.94\times {10}^{-15}$, ratio = 0.376), Cell Cycle Control of Chromosomal Replication (adj. Pval. $=5.89\times {10}^{-09}$, ratio = 0.393), Role of BRCA1 in DNA Damage Response (adj. Pval. $=1.32\times {10}^{-08}$, ratio = 0.325) and IL-8 Signaling (adj. Pval. $=4.37\times {10}^{-08}$, ratio = 0.215) as some of the top dysregulated pathways. More importantly, major aberrant pathways shown to be involved in GBM pathogenesis [36, 37] were also identified and include Glioblastoma Multiforme Signaling (adj. Pval. $=2.95\times {10}^{-06}$, ratio = 0.206), Glioma Signaling (adj. Pval. $=3.63\times {10}^{-05}$, ratio = 0.205), p53 Signaling (adj. Pval. $=5.25\times {10}^{-05}$, ratio = 0.224), Glioma Invasiveness Signaling (adj. Pval. $=0.0008$, ratio = 0.219), PI3K/AKT Signaling (adj. Pval. $=0.005$, ratio = 0.146) and mTOR Signaling (adj. Pval. $=0.007$, ratio = 0.138).

**Fig. 5:**
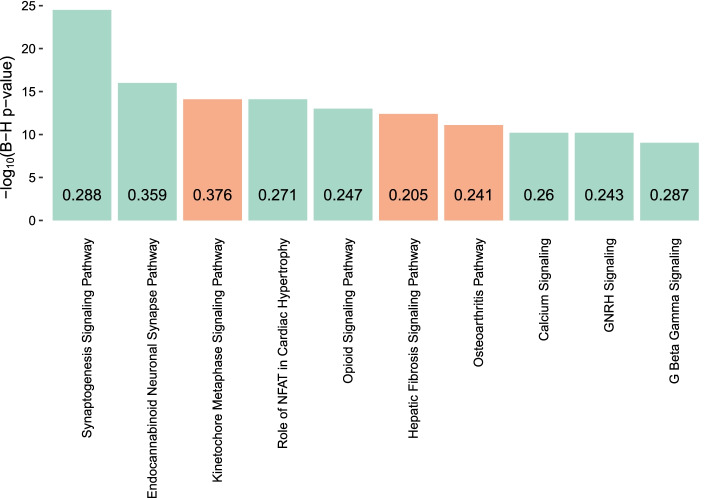
Significant pathways identified by IPA. The top ten significant pathways based on Benjamini Hochberg (BH) *p*-value among the canonical pathways identified by Ingenuity Pathway Analysis (IPA) for the up-regulated differentially expressed genes (DEGs) (orange bar) and down-regulated DEGs (green bar). The numbers on the bar plot show the ratio between the numbers of DEGs enriched and total number of genes in each of these pathways

Similarly, all 1845 down-regulated DEGs mapped to the IPA database and 88 canonical pathways were identified as significant (BH adjusted *p*-value < 0.01). Synaptogenesis Signaling Pathway (adj. Pval. $=3.16\times {10}^{-25}$, ratio = 0.288), Endocannabinoid Neuronal Synapse Pathway (adj. Pval. $=1.00\times {10}^{-16}$, ratio = 0.359), Opioid Signaling Pathway (adj. Pval. $=1.00\times {10}^{-13}$, ratio = 0.247), GNRH Signaling (adj. Pval. $=6.31\times {10}^{-11}$, ratio = 0.260), Calcium Signaling (adj. Pval. $=6.31\times {10}^{-11}$, ratio = 0.243), G Beta Gamma Signaling (adj. Pval. $=9.33\times {10}^{-10}$, ratio = 0.287) and Dopamine-DARPP32 Feedback in cAMP Signaling (adj. Pval. $=1.55\times {10}^{-09}$, ratio = 0.252) were identified as some of the top dysregulated pathways. The top 10 pathways identified by the up-regulated and down-regulated DEGs separately are illustrated in Fig. 5. For complete list of identified pathways for up- and down-regulated DEGs in our study, see Additional file 1: Table S7.

In addition, the URA tool in IPA identified potential upstream regulators (transcription factors, genes or other small molecules) that has been experimentally observed to affect gene expression. It identifies these regulators by analysing linkage to DEGs through coordinated expression [38]. Among the up-regulated DEGs, *TGFB1* and *TP53*, which are also DEGs and important in GBM pathogenesis [39, 40] are predicted to be the top two upstream regulators. 293 up-regulated DEGs were identified as potential upstream regulators of gene upregulation out of a total of 2215 (BH corrected *p*-value < 0.01, see Additional file 1: Table S8a) predicted upstream regulators. Out of 2215 predicted, 764 of these significant upstream regulators were activated and 112 were also observed as DEGs in our analysis.

On the contrary, for the down-regulated DEGs, IPA identified 32 potential upstream regulators (BH corrected *p*-value < 0.01, see Additional file 1: Table S8b) with *TCF7L2* and *MAPT* as the top two. 14 of the 32 upstream regulated were predicted to be inhibited and two among the inhibited are DEGs. *TCF7L2* is a diabetes risk-associated gene which plays a key role in the Wnt-signaling pathway and is shown to be frequently mutated in colorectal cancer [41] and promote cell proliferation [42]. However, exploration of its role in GBM pathogenesis warrant further studies. Interestingly, *MAPT* is also a DEG observed in our analysis and is one of the two hallmarks of AD [43]. Gargini et al. [44] observed a strong correlation of Tau/MAPT expression and indicators of survival in glioma patients. Moreover, it has been found to be epigenetically controlled by balance between *IDH1/2* wild-type and mutation in human gliomas [45]. Thus, providing further evidence and reaffirming the involvement of *MAPT* in central nervous system disorders.

Of the DEGs with conflicting direction of expression across studies, 182 out of 235 DEGs are present in all three datasets. Among them *CMTM6*, *RAD51*, *NOS1AP*, *MSANTD1*, *PGM2*, *PSD3*, *GPR82*, *SPTBN4*, *TSPAN6* and *ARHGEF28* were identified as top 10 DEGs based on the absolute value of ${N}_{g}$ (see Table 5). Interestingly, *RAD51* and *ARHGEF28* have previously been identified as a tumour suppressor and an oncogene respectively [46]. More importantly, *RAD51* was found to be effectively over-expressed in GBM in our study and have recently been shown as a target for inhibition to enhance radiosensitivity of GBM cells during treatment [47, 48]. On the other hand, *ARHGEF28* was found to be effectively down-regulated in our study. It is an intracellular kinase that functions either as a Rho guanine exchange factor or a scaffolding protein to initiate FAK activation and cell contractibility [49]. Furthermore, the RhoA-FAK pathway has been shown to be involved in colon cancer cell proliferation and migration [50]. *ARHGEF28* mRNA levels have also been found to be elevated in late-stage ovarian cancer and associated with decreased progression free and overall survival [51]. However, its role in GBM growth and progression is yet to be elucidated and requires exploration in future studies.

**Table 5:** Top 10 differentially expressed genes (DEGs) with mismatched direction of expression across datasets identified by the fused inverse-normal method

DEGs	${N}_{g}$	Mean |logFC|	Effect	BH *p*-value
*CMTM6*	7.58	1.30	+−+	$3.93\times {10}^{-13}$
*RAD51*	7.58	2.82	+−+	$4.03\times {10}^{-13}$
*NOS1AP*	− 7.53	1.37	−+−	$5.73\times {10}^{-13}$
*MSANTD1*	− 7.53	1.31	−+−	$5.92\times {10}^{-13}$
*PGM2*	7.52	1.31	+−+	$6.35\times {10}^{-13}$
*PSD3*	− 7.47	1.67	−+−	$8.63\times {10}^{-13}$
*GPR82*	7.43	4.35	+−+	$1.24\times {10}^{-12}$
*SPTBN4*	− 7.31	1.91	−+−	$2.78\times {10}^{-12}$
*TSPAN6*	7.18	2.08	+−+	$6.86\times {10}^{-12}$
*ARHGEF28*	− 7.06	1.23	+−−	$1.63\times {10}^{-11}$

The DEGs have been sorted based on the absolute value of the statistic ${N}_{g}$ and the mean of absolute value of the ${\mathrm{log}}_{2}FC$ have been reported. Effect signifies the direction of expression of DEGs in the per-study differential analysis for GSE123892, GSE151352 and TCGA-GBM respectively. BH *p*-value: Benjamini Hochberg *p*-value

## Discussion

Although the implementation of MIN and FIN *p*-value combination methods are straight forward, they require some additional considerations. First, the used weighting criteria leads to a larger weight being given to a study with larger sample sizes. Intuitively, this is expected as a study with a larger sample size might be more robust than studies with lower sample sizes. However, importance must also be given to the quality of the RNA-seq data in each study. It must be assessed in case this information is available and other weights more appropriate as per the quality of the data may be specified.

Next, the MIN and FIN are adaptive in a sense that they allow for consideration of genes that may not be present in all studies that are considered for integrated differential expression analysis. In case a gene is not present in some of the studies, the weights (${w}_{s}$) in the combination method can only be estimated using the number of replicates in the datasets in which the gene is present. However, for genes that are just present in one study, it would mean that the results from the meta-analysis for these genes would be the same as the per-study differential analysis. Hence, a careful consideration about the quality of the RNA-seq data and library size is required in case only the genes that are common among studies are considered. For datasets of similar quality and library size, a large proportion of genes would not be excluded from meta-analysis if only common genes are used. However, a large number of genes might be excluded from meta-analysis in case of dissimilar library sizes and quality which could lead to potentially missing out on important genes for the disease. For instance, only 12,345 out of 18,315 unique genes are present in all 3 studies in our application where the library sizes are not similar. Thus, a balanced approach is suggested.

Finally, we used edgeR for per-study differential analysis in our study but other popular packages such as DESeq2 [52] and NOIseq [35] can be applied. Moreover, the FIN model can be extended to multi-group comparisons apart from a two-group comparison discussed in this study. The proposed meta-analysis method relies on the fact that the same test statistics are used for per-study differential expression analysis to obtain individual *p*-values and all studies under consideration have the same experimental considerations. For instance, in case DESeq2 is used for multi-group differential expression analysis in each study, a likelihood ratio test is used rather than Wald statistics being used for two group differential expression analysis.

## Conclusions

In this study, we proposed MIN and consequently FIN method for meta-analysis of RNA-seq data. The developed methods account for both the sample size of study and direction of expression of a gene in each study allowing for detection of potentially robust biologically significant DEGs even when they have conflicting direction of expression across studies. In contrast with the existing IN method, the proposed methods have the advantage of identifying DEGs among genes with conflicting direction of expression across studies. For the genes with concordant differential expression patterns across studies the MIN method exhibited a similar DEG detection power and performance as compared to IN method particularly when there was high inter-study variability and increased number of studies were considered. FIN method exhibited a similar or improved DEG detection power as compared to IN method and was significantly better in performance as compared to MIN method. More importantly, in a real data application, we demonstrated the use of FIN method in detection of biologically relevant DEGs to GBM. Hence, this meta-analysis method provides a way to establish differential expression status for genes with conflicting direction of expression in individual RNA-seq studies and further exploration of them as potential biomarkers for the disease. With lowering costs and increase in the number of RNA-seq studies being archived on public databases, this method might provide a way to integrate a greater number of studies without losing much prior information and consequently considering all the genes in the analysis irrespective of their direction of expression.

## Supplementary Information

**Additional file 1**. Document contains interpretation of the fused inverse-normal (FIN) method, details of RNA-seq raw data processing using GALAXY, brief description of the simulation method, results of scenario 2 considered for meta-analysis and Ingenuity pathway analysis results.

**Additional file 2**. Full list of differentially expressed genes (DEGs) identified in scenario 1 by the inverse-normal (IN) method in glioblastoma (GBM) data application.

**Additional file 3**. Full list of differentially expressed genes (DEGs) identified in scenario 1 by the modified inverse-normal (MIN) method in glioblastoma (GBM) data application.

**Additional file 4**. Full list of differentially expressed genes (DEGs) identified in scenario 1 by the fused inverse-normal (FIN) method in glioblastoma (GBM) data application.

## Abbreviations

RNA-seq: RNA sequencing
DEGs: Differentially expressed genes
GBM: Glioblastoma
IN: Inverse-normal
PANDORA: PerformANce Driven scOring of RNA-Seq stAtistics
MIN: Modified inverse-normal
FIN: Fused inverse-normal
IPA: Ingenuity pathway analysis
*H*_0_: Null hypothesis
FDR: False discovery rate
BH: Benjamini–Hochberg
AD: Alzheimer’s disease
GEO: Gene expression omnibus
ROC: Receiver operating characteristic
AUC: Area under curve
TCGA: The Cancer Genome Atlas
CPM: Counts per million
TMM: Trimmed mean of M-values
URA: Upstream regulator analysis
TP: True positives
RAD51: RAD51 recombinase
TCF7L2: Transcription factor 7 like 2
MAPT: Microtubule associated protein tau
TGFB1: Transforming growth factor beta 1
TP53: Tumor protein 53
IDH1/2: Isocitrate dehydrogenase 1/2
CMTM6: CKLF like MARVEL transmembrane domain containing 6
NOS1AP: Nitric oxide synthase 1 adaptor protein
MSANTD1: Myb/SANT DNA binding domain containing 1
PGM2: Phosphoglucomutase 2
PSD3: Pleckstrin and Sec7 domain containing 3
GPR82: G protein-coupled receptor 82
SPTBN4: Spectrin beta, non-erythrocytic 4
TSPAN6: Tetraspanin 6
ARHGEF28: Rho guanine nucleotide exchange factor 28

## Declarations

### Availability of data and materials

The datasets analysed during the current study are available on Gene Expression Omnibus (GEO) and The Cancer Genome Atlas (TCGA) public repository. RNA-seq datasets used and available on Gene Expression Omnibus (GEO) can be found using accession numbers GSE123892, GSE151352 and GSE125583. TCGA-GBM RNA-seq dataset can be accessed here: https://portal.gdc.cancer.gov/projects/TCGA-GBM. An implementation of the proposed method can be found here: https://github.com/nash5202/FIN_meta_analysis/.
