# Supplementary material for: Fused inverse-normal method for integrated differential expression analysis of RNA-seq data
Source: BMC Bioinformatics. 2022 Aug 5;23:320. doi: 10.1186/s12859-022-04859-9 (PMC9354357; doi:10.1186/s12859-022-04859-9)
Supplement: Supplementary file 1 — Additional file 1. Document contains interpretation of the fused inverse-normal (FIN) method, details of RNA-seq raw data processing using GALAXY, brief description of the simulation method, results of scenario 2 considered for meta-analysis and Ingenuity pathway analysis results. [file 12859_2022_4859_MOESM1_ESM.docx]

**Fused inverse-normal method for integrated differential expression analysis of RNA-seq data**

Birbal Prasad^1^ and Xinzhong Li^1*^

^1^National Horizons Centre, School of Health and Life Sciences, Teesside University, Darlington, DL1 1HG, UK. Email: [B.Prasad@tees.ac.uk](mailto:B.Prasad@tees.ac.uk); [X.Li@tees.ac.uk](mailto:X.Li@tees.ac.uk)

*Correspondence: Email: [X.Li@tees.ac.uk](mailto:X.Li@tees.ac.uk), Phone: +44-01642738451

**Supplementary information contents lists:**

1. **Interpretations of the FIN method**
2. **Simulation study model**
3. **Processing of raw RNA-seq dataset GSE151352 using GALAXY**
4. **Supplementary Figure 1.** Flow of different steps used in processing of the raw RNA-seq fastq files for GSE151352 using Galaxy to get raw counts.
5. **Supplementary Table 1.** Total number of genes (# genes), number of genes with conflicting direction of expression (# conf. genes), number of differentially expressed genes (# DEGs), the number of differentially expressed genes with conflicting direction of expression (# conf. DEGs) and number of true differentially expressed genes (# true DEGs) for all simulation settings averaged over 100 trials.
6. **Supplementary Table 2.** Total number of up and down-regulated differentially expressed genes (DEGs) identified in per-study differential analysis in 10 random selections of 20 glioblastoma (GBM) cases and 5 controls from TCGA-GBM dataset.
7. **Supplementary Table 3.** Number of common differentially expressed genes (DEGs) (between scenario 1 and 2) identified by the fused inverse-normal (FIN) method. On average about 94% of the DEGs obtained when randomly selected subset was considered (scenario 2) were also found in DEGs identified using the full TCGA-GBM dataset (scenario1). Total #DEGs: Total number of differentially expressed genes in scenario 2 identified by the FIN method.
8. **Supplementary Table 4.** Number of differentially expressed genes (DEGs) identified by the inverse-normal (IN) method in the glioblastoma (GBM) data application for both scenarios and number of these DEGs present in one, two or all three datasets considered.
9. **Supplementary Table 5.** Number of differentially expressed genes (DEGs) identified by the modified inverse-normal (MIN) method in the glioblastoma (GBM) data application for both scenarios and number of these DEGs present in one, two or all three datasets considered. **a.** For DEGs with the same direction of expression across all three studies. **b.** For DEGs with conflicting direction of expression across studies.
10. **Supplementary Table 6.** Number of differentially expressed genes (DEGs) identified by the fused inverse-normal (FIN) method in the glioblastoma (GBM) data application for both scenarios and number of these DEGs present in one, two or all three datasets considered. **a.** For DEGs with the same direction of expression across all three studies. **b.** For DEGs with conflicting direction of expression across studies
11. **Supplementary Table 7.** Ingenuity Pathway Analysis (IPA) canonical pathways for **a.** up and **b.** down-regulated differentially expressed genes (DEGs) present in all three glioblastoma (GBM) datasets and identified by the fused inverse-normal (FIN) method. Ratio denotes the number of DEGs enriched in a pathway to the total number of genes in that pathway.
12. **Supplementary Table 8.** Ingenuity Pathway Analysis (IPA) upstream regulator analysis results for **a.** up and **b.** down-regulated differentially expressed genes (DEGs) present in all three glioblastoma (GBM) datasets and identified by the fused inverse-normal (FIN) method.
13. **Supplementary 2.** Full list of differentially expressed genes (DEGs) identified in scenario 1 by the inverse-normal (IN) method in glioblastoma (GBM) data application (in an Excel file).
14. **Supplementary 3.** Full list of differentially expressed genes (DEGs) identified in scenario 1 by the modified inverse-normal (MIN) method in glioblastoma (GBM) data application (in an Excel file).
15. **Supplementary 4.** Full list of differentially expressed genes (DEGs) identified in scenario 1 by the fused inverse-normal (FIN) method in glioblastoma (GBM) data application (in an Excel file).

**Interpretations of the FIN method**

To understand the practical behaviour and interpretations of the FIN method in different scenarios, we consider the following cases in terms of differential expression of a gene and its direction of expression (up: +, down: -) in individual studies:

1. A gene is non differentially expressed (NDE) in one study and differentially expressed (DE) in another: If it is NDE, $\Phi^{-1}\left( 1-p_{gs} \right)$ is standard normal distributed. Hence, it takes values around 0 or small values with high probability. On the contrary, if it is DE, $\Phi^{-1}\left( 1-p_{gs} \right)$ will take large values. Now, the value of $N_{g}$ will then depend on which study has the larger weight ($w_{s}$).
2. A gene is strongly +DE in one study and strongly -DE in another: In this case, $\Phi^{-1}\left( 1-p_{gs} \right)$ will be large values for both studies. Then, if the weight $w_{s}$ is similar for both studies, the contribution of the study specific term to $N_{g}$ will cancel each other out because of $B_{gs}$. Hence, $N_{g}$ which will be close to 0 will give us non-significant p-value in hypothesis testing for that gene. This makes sense as we will have comparable evidence of conflicting direction of expression for the gene. In case, the weight of one study is comparatively larger than the other study, we will get that the gene is DE with the effective sign of regulation of that of the bigger study.
3. A gene is strongly +DE in most studies and weakly -DE in other few: Similar to case ii, $\Phi^{-1}\left( 1-p_{gs} \right)$ will be large for all the studies. For comparable weights of studies, we would get a large positive value for $N_{g}$ as we have many more studies where the gene is +DE as compared to where the gene is -DE. Hence, resulting in the gene to be +DE after hypothesis testing. Only in case when the studies where the gene is -DE has extremely large weights $w_{s}$ as compared to all other +DE studies combined can that gene be -DE in hypothesis testing.

**Simulation study model**

Here, we briefly describe the theoretical framework of the simulation study method adapted from Rau et al. (2014) [reference 10 in the manuscript]. For detailed procedure for estimation of parameter values based on real RNA-seq datasets (GSE125583 in this study), see Rau et al. (2014).

Let $Y_{gcrs}$ be random variable with $y_{gcrs}$ as its realisations which are observed count for a gene $g$, condition $c$, biological replicate $r$ and study $s$. RNA-seq data was generated as per the negative binomial distribution,

$$Y_{gcrs}\sim NB(\mu_{gcs}, \phi_{gs})$$

where parameters $\mu_{gcs}$ and $\phi_{gs}$ represent the mean and dispersion, respectively, for a gene $g$, condition $c$ and study $s$. To account for variability between the individual studies (inter-study variability) considered for meta-analysis, we consider the following situation:

$$\mu_{gcs}=\theta_{gc}\times e^{\epsilon_{gcs}}$$

where $\epsilon_{gcs}\sim N\left( 0, \sigma^{2} \right)$. $\theta_{gc}$ is the mean for a gene $g$ in condition $c$. $\epsilon_{gcs}$ represents the variability around $\theta_{gc}$ due to a study and condition specific random effect. $\sigma^{2}$ represents the size of the inter-study variability which affects $\mu_{gcs}$ through $\epsilon_{gcs}$ with $\epsilon_{gcs}$ having a multiplicative effect on $\mu_{gcs}$.

**Processing of raw RNA-seq dataset GSE151352 using GALAXY**

Raw fastq files generated by GPL23934 (Ion Torrent S5 (Homo sapiens)) platform were retrieved for all 24 samples (12 tumour and 12 healthy) from Sequence Read Archive (<https://trace.ncbi.nlm.nih.gov/Traces/sra/sra.cgi?study=SRP265074>) using Get Data tool in Galaxy. Next, we assessed the quality of the raw reads using quality reports from FastQC and removed the specified adapter sequence ATCACCGACTGCCCATAGAGAGGCTGAGAC with Cutadapt (version 1.16). The parameters used for this step were the parameters provided by the submitted of the dataset on Gene Expression Omnibus. The adapter trimmed reads were again assessed for quality by FastQC and were aligned to the reference genome (GRCh37.p13, genecode v19) using a 2-pass method with RNA STAR (Galaxy version 2.7.5b) where other parameters used were default parameters. Following alignment, the generated BAM files were processed using the featureCounts tool (Galaxy version 1.6.4+galaxy2) to get raw counts for each RNA-seq data sample.


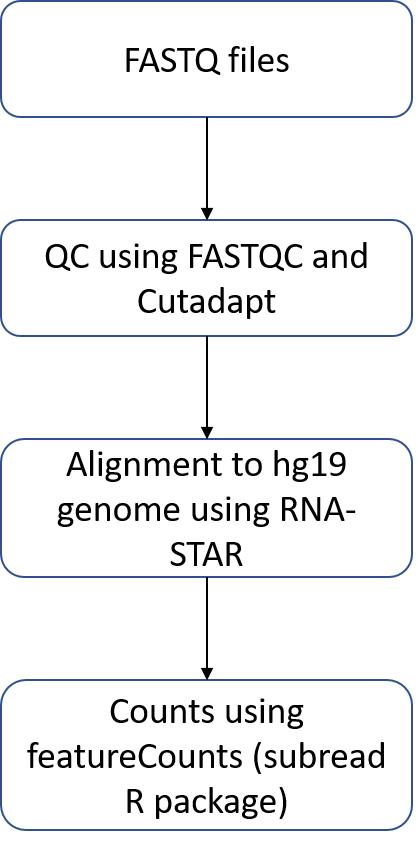


**Figure 1.**  Flow of different steps used in processing of the raw RNA-seq fastq files for GSE151352 using Galaxy to get raw counts.

**Supplementary Table 1.** Total number of genes (# genes), number of genes with conflicting direction of expression (# conf. genes), number of differentially expressed genes (# DEGs), the number of differentially expressed genes with conflicting direction of expression (# conf. DEGs) and number of true differentially expressed genes (# true DEGs) for all simulation settings averaged over 100 trials.

| Setting  ($\boldsymbol{\sigma}$, #studies) | # genes  (IN/MIN/FIN) | # conf. genes  (IN/MIN/FIN) | # DEGs  (IN, MIN, FIN) | # conf. DEGs  (IN, MIN, FIN) | # true DEGs |
| --- | --- | --- | --- | --- | --- |
| 0.15, 3 | 16776 ± 19 | 10099 ± 49 | 2268 ± 38, 2611 ± 49, 2441 ± 45 | 0, 243 ± 18, 236 ± 18 | 6436 ± 10 |
| 0.15, 5 | 16871 ± 17 | 13036 ± 43 | 2431 ± 35, 4031 ± 47, 3741 ± 45 | 0, 1365 ± 39, 1337 ± 38 | 6468 ± 9 |
| 0.5, 3 | 17002 ± 45 | 11490 ± 57 | 2098 ± 35, 3879 ± 46, 3741 ± 44 | 0, 1706 ± 35, 1697 ± 35 | 6501 ± 19 |
| 0.5, 5 | 17266 ± 34 | 14824 ± 48 | 1124 ± 31, 4453 ± 48, 4365 ± 46 | 0, 3264 ± 46, 3257 ± 45 | 6585 ± 15 |

**Supplementary Table 2.** Total number of up and down-regulated differentially expressed genes (DEGs) identified in per-study differential analysis in 10 random selections of 20 glioblastoma (GBM) cases and 5 controls from TCGA-GBM dataset.

| TCGA-GBM | DEGs (Up) | DEGs (Down) | Total |
| --- | --- | --- | --- |
| Selection 1 | 3112 | 3016 | 6128 |
| Selection 2 | 3323 | 3013 | 6336 |
| Selection 3 | 3204 | 3030 | 6234 |
| Selection 4 | 3285 | 3054 | 6339 |
| Selection 5 | 3267 | 3077 | 6344 |
| Selection 6 | 3249 | 3069 | 6318 |
| Selection 7 | 3293 | 2968 | 6261 |
| Selection 8 | 3792 | 2885 | 6677 |
| Selection 9 | 3311 | 3025 | 6336 |
| Selection 10 | 3229 | 3088 | 6317 |

**Supplementary Table 3.** Number of common differentially expressed genes (DEGs) (between scenario 1 and 2) identified by the fused inverse-normal (FIN) method. On average about 94% of the DEGs obtained when randomly selected subset was considered (scenario 2) were also found in DEGs identified using the full TCGA- GBM dataset (scenario1). Total #DEGs: Total number of differentially expressed genes in scenario 2 identified by the FIN method.

| Scenario 2 | Common DEGs | Total # DEGs | %common |
| --- | --- | --- | --- |
| Selection 1 | 5339 | 5665 | 94.25 |
| Selection 2 | 5406 | 5697 | 94.89 |
| Selection 3 | 5308 | 5645 | 94.03 |
| Selection 4 | 5382 | 5710 | 94.26 |
| Selection 5 | 5358 | 5722 | 93.64 |
| Selection 6 | 5336 | 5743 | 92.91 |
| Selection 7 | 5382 | 5684 | 94.69 |
| Selection 8 | 5400 | 5797 | 93.15 |
| Selection 9 | 5352 | 5720 | 93.57 |
| Selection 10 | 5415 | 5698 | 95.03 |

**Supplementary Table 4.** Number of differentially expressed genes (DEGs) identified by the inverse-normal (IN) method in the glioblastoma (GBM) data application for both scenarios and number of these DEGs present in one, two or all three datasets considered.

| Datasets | DEGs  Present in 1 | DEGs  Present in 2 | DEGs  Present in 3 |
| --- | --- | --- | --- |
| GSE123892, GSE151352, TCGA-GBM (all samples) | 1368 | 1085 | 3465 |
| GSE123892, GSE151352, TCGA-GBM (random selection 1) | 921 | 1082 | 3511 |
| GSE123892, GSE151352, TCGA-GBM (random selection 2) | 943 | 1080 | 3477 |
| GSE123892, GSE151352, TCGA-GBM (random selection 3) | 939 | 1079 | 3462 |
| GSE123892, GSE151352, TCGA-GBM (random selection 4) | 965 | 1080 | 3482 |
| GSE123892, GSE151352, TCGA-GBM (random selection 5) | 939 | 1076 | 3540 |
| GSE123892, GSE151352, TCGA-GBM (random selection 6) | 940 | 1100 | 3543 |
| GSE123892, GSE151352, TCGA-GBM (random selection 7) | 972 | 1055 | 3461 |
| GSE123892, GSE151352, TCGA-GBM (random selection 8) | 989 | 1106 | 3513 |
| GSE123892, GSE151352, TCGA-GBM (random selection 9) | 921 | 1083 | 3529 |
| GSE123892, GSE151352, TCGA-GBM (random selection 10) | 995 | 1080 | 3442 |

**Supplementary Table 5.** Number of differentially expressed genes (DEGs) identified by the modified inverse-normal (MIN) method in the glioblastoma (GBM) data application for both scenarios and number of these DEGs present in one, two or all three datasets considered. **a.** For DEGs with the same direction of expression across all three studies. **b.** For DEGs with conflicting direction of expression across studies.

| 1. For DEGs with the same direction of expression across all 3 studies | | | | |
| --- | --- | --- | --- | --- |
| Datasets | **DEGs**  **Present in 1** | | **DEGs**  **Present in 2** | **DEGs**  **Present in 3** |
| GSE123892, GSE151352, TCGA-GBM (all samples) | 1182 | 1087 | | 3623 |
| GSE123892, GSE151352, TCGA-GBM (random selection 1) | 832 | 1081 | | 3658 |
| GSE123892, GSE151352, TCGA-GBM (random selection 2) | 844 | 1099 | | 3662 |
| GSE123892, GSE151352, TCGA-GBM (random selection 3) | 861 | 1089 | | 3616 |
| GSE123892, GSE151352, TCGA-GBM (random selection 4) | 862 | 1088 | | 3654 |
| GSE123892, GSE151352, TCGA-GBM (random selection 5) | 845 | 1078 | | 3695 |
| GSE123892, GSE151352, TCGA-GBM (random selection 6) | 863 | 1107 | | 3689 |
| GSE123892, GSE151352, TCGA-GBM (random selection 7) | 876 | 1062 | | 3644 |
| GSE123892, GSE151352, TCGA-GBM (random selection 8) | 887 | 1094 | | 3697 |
| GSE123892, GSE151352, TCGA-GBM (random selection 9) | 830 | 1097 | | 3702 |
| GSE123892, GSE151352, TCGA-GBM (random selection 10) | 911 | 1090 | | 3605 |

| 1. For DEGs with the mismatched direction of expression across 3 studies | | | | |
| --- | --- | --- | --- | --- |
| Datasets | **DEGs**  **Present in 1** | | **DEGs**  **Present in 2** | **DEGs**  **Present in 3** |
| GSE123892, GSE151352, TCGA-GBM (all samples) | 0 | 52 | | 181 |
| GSE123892, GSE151352, TCGA-GBM (random selection 1) | 0 | 26 | | 140 |
| GSE123892, GSE151352, TCGA-GBM (random selection 2) | 0 | 41 | | 171 |
| GSE123892, GSE151352, TCGA-GBM (random selection 3) | 0 | 38 | | 149 |
| GSE123892, GSE151352, TCGA-GBM (random selection 4) | 0 | 34 | | 166 |
| GSE123892, GSE151352, TCGA-GBM (random selection 5) | 0 | 28 | | 154 |
| GSE123892, GSE151352, TCGA-GBM (random selection 6) | 0 | 33 | | 140 |
| GSE123892, GSE151352, TCGA-GBM (random selection 7) | 0 | 39 | | 173 |
| GSE123892, GSE151352, TCGA-GBM (random selection 8) | 0 | 30 | | 179 |
| GSE123892, GSE151352, TCGA-GBM (random selection 9) | 0 | 39 | | 165 |
| GSE123892, GSE151352, TCGA-GBM (random selection 10) | 0 | 34 | | 164 |

**Supplementary Table 6.** Number of differentially expressed genes (DEGs) identified by the fused inverse-normal (FIN) method in the glioblastoma (GBM) data application for both scenarios and number of these DEGs present in one, two or all three datasets considered. **a.** For DEGs with the same direction of expression across all three studies. **b.** For DEGs with conflicting direction of expression across studies.

| 1. For DEGs with the same direction of expression across all 3 studies | | | | |
| --- | --- | --- | --- | --- |
| Datasets | **DEGs**  **Present in 1** | | **DEGs**  **Present in 2** | **DEGs**  **Present in 3** |
| GSE123892, GSE151352, TCGA-GBM (all samples) | 1359 | 1083 | | 3461 |
| GSE123892, GSE151352, TCGA-GBM (random selection 1) | 911 | 1080 | | 3508 |
| GSE123892, GSE151352, TCGA-GBM (random selection 2) | 933 | 1077 | | 3475 |
| GSE123892, GSE151352, TCGA-GBM (random selection 3) | 931 | 1070 | | 3457 |
| GSE123892, GSE151352, TCGA-GBM (random selection 4) | 957 | 1077 | | 3476 |
| GSE123892, GSE151352, TCGA-GBM (random selection 5) | 931 | 1073 | | 3536 |
| GSE123892, GSE151352, TCGA-GBM (random selection 6) | 935 | 1096 | | 3540 |
| GSE123892, GSE151352, TCGA-GBM (random selection 7) | 962 | 1051 | | 3460 |
| GSE123892, GSE151352, TCGA-GBM (random selection 8) | 978 | 1101 | | 3509 |
| GSE123892, GSE151352, TCGA-GBM (random selection 9) | 912 | 1078 | | 3526 |
| GSE123892, GSE151352, TCGA-GBM (random selection 10) | 988 | 1076 | | 3436 |

| 1. For DEGs with the mismatched direction of expression across 3 studies | | | | |
| --- | --- | --- | --- | --- |
| Datasets | **DEGs**  **Present in 1** | | **DEGs**  **Present in 2** | **DEGs**  **Present in 3** |
| GSE123892, GSE151352, TCGA-GBM (all samples) | 0 | 53 | | 182 |
| GSE123892, GSE151352, TCGA-GBM (random selection 1) | 0 | 26 | | 140 |
| GSE123892, GSE151352, TCGA-GBM (random selection 2) | 0 | 41 | | 171 |
| GSE123892, GSE151352, TCGA-GBM (random selection 3) | 0 | 38 | | 149 |
| GSE123892, GSE151352, TCGA-GBM (random selection 4) | 0 | 34 | | 166 |
| GSE123892, GSE151352, TCGA-GBM (random selection 5) | 0 | 28 | | 154 |
| GSE123892, GSE151352, TCGA-GBM (random selection 6) | 0 | 32 | | 140 |
| GSE123892, GSE151352, TCGA-GBM (random selection 7) | 0 | 39 | | 172 |
| GSE123892, GSE151352, TCGA-GBM (random selection 8) | 0 | 30 | | 179 |
| GSE123892, GSE151352, TCGA-GBM (random selection 9) | 0 | 39 | | 165 |
| GSE123892, GSE151352, TCGA-GBM (random selection 10) | 0 | 34 | | 164 |

**Supplementary Table 7.** Ingenuity Pathway Analysis (IPA) canonical pathways for **a.** up and **b.** down-regulated differentially expressed genes (DEGs) present in all three glioblastoma (GBM) datasets and identified by the fused inverse-normal (FIN) method. Ratio denotes the number of DEGs enriched in a pathway to the total number of genes in that pathway.

| **Ingenuity Canonical Pathways** | **-log(BH p-value)** | **Ratio** | **z-score** |
| --- | --- | --- | --- |
| 1. **Pathways from up-regulated DEGs** | | | |
| Kinetochore Metaphase Signaling Pathway | 14.1 | 0.376 | 3.413 |
| Hepatic Fibrosis Signaling Pathway | 12.4 | 0.205 | 7.714 |
| Osteoarthritis Pathway | 11.1 | 0.241 | 5.126 |
| Cell Cycle Control of Chromosomal Replication | 8.23 | 0.393 | 4.69 |
| Role of BRCA1 in DNA Damage Response | 7.88 | 0.325 | 2.837 |
| IL-8 Signaling | 7.36 | 0.215 | 5.84 |
| Tumor Microenvironment Pathway | 6.56 | 0.216 | 4.867 |
| Estrogen-mediated S-phase Entry | 6.32 | 0.5 | 2.496 |
| Death Receptor Signaling | 6.19 | 0.272 | 3.4 |
| GP6 Signaling Pathway | 6.17 | 0.244 | 5.385 |
| Acute Phase Response Signaling | 5.98 | 0.206 | 4.596 |
| Colorectal Cancer Metastasis Signaling | 5.9 | 0.182 | 5.376 |
| Dendritic Cell Maturation | 5.76 | 0.201 | 5.831 |
| Glioblastoma Multiforme Signaling | 5.53 | 0.206 | 4.158 |
| ILK Signaling | 5.43 | 0.195 | 4.7 |
| FAT10 Cancer Signaling Pathway | 5.43 | 0.348 | 2 |
| EIF2 Signaling | 5.35 | 0.183 | 2.711 |
| Senescence Pathway | 5.32 | 0.171 | 1.021 |
| Type I Diabetes Mellitus Signaling | 5.27 | 0.234 | 3.441 |
| Regulation Of The Epithelial Mesenchymal Transition By Growth Factors Pathway | 5.16 | 0.191 | 5.667 |
| Production of Nitric Oxide and Reactive Oxygen Species in Macrophages | 5.12 | 0.19 | 5.24 |
| Neuroinflammation Signaling Pathway | 5.03 | 0.163 | 6.112 |
| HOTAIR Regulatory Pathway | 5.01 | 0.2 | 3.772 |
| Cyclins and Cell Cycle Regulation | 5 | 0.259 | 1.528 |
| Signaling by Rho Family GTPases | 4.87 | 0.17 | 5.745 |
| Role of PKR in Interferon Induction and Antiviral Response | 4.84 | 0.22 | 3.411 |
| Leukocyte Extravasation Signaling | 4.58 | 0.181 | 3.889 |
| Pancreatic Adenocarcinoma Signaling | 4.5 | 0.22 | 2.84 |
| Glioma Signaling | 4.44 | 0.218 | 3.317 |
| HIF1α Signaling | 4.43 | 0.176 | 4.333 |
| Tec Kinase Signaling | 4.39 | 0.185 | 4.796 |
| p53 Signaling | 4.28 | 0.224 | 0.535 |
| Inhibition of Angiogenesis by TSP1 | 4.26 | 0.353 | 2.53 |
| Regulation of Cellular Mechanics by Calpain Protease | 3.95 | 0.243 | 1.732 |
| IL-6 Signaling | 3.94 | 0.198 | 4.491 |
| STAT3 Pathway | 3.87 | 0.193 | 2.982 |
| Bladder Cancer Signaling | 3.86 | 0.216 | 2 |
| TNFR1 Signaling | 3.76 | 0.28 | 3.051 |
| Ovarian Cancer Signaling | 3.67 | 0.187 | 3.317 |
| Apoptosis Signaling | 3.67 | 0.21 | 1.789 |
| BEX2 Signaling Pathway | 3.6 | 0.228 | 2.828 |
| Breast Cancer Regulation by Stathmin1 | 3.57 | 0.125 | 6.215 |
| Mitotic Roles of Polo-Like Kinase | 3.54 | 0.242 | 1.069 |
| MSP-RON Signaling In Cancer Cells Pathway | 3.54 | 0.187 | 4.6 |
| Aryl Hydrocarbon Receptor Signaling | 3.51 | 0.182 | 0.894 |
| Systemic Lupus Erythematosus In B Cell Signaling Pathway | 3.47 | 0.149 | 4.841 |
| ATM Signaling | 3.43 | 0.206 | 0.775 |
| TREM1 Signaling | 3.42 | 0.227 | 4.123 |
| Induction of Apoptosis by HIV1 | 3.42 | 0.246 | 2.84 |
| NF-κB Signaling | 3.42 | 0.168 | 4.747 |
| Notch Signaling | 3.25 | 0.297 | 1.414 |
| Myc Mediated Apoptosis Signaling | 3.23 | 0.26 | 2.496 |
| Sphingosine-1-phosphate Signaling | 3.21 | 0.188 | 1.706 |
| Fcγ Receptor-mediated Phagocytosis in Macrophages and Monocytes | 3.18 | 0.202 | 4.359 |
| Glioma Invasiveness Signaling | 3.09 | 0.219 | 2.5 |
| Th2 Pathway | 3.09 | 0.176 | 1.732 |
| Crosstalk between Dendritic Cells and Natural Killer Cells | 3.04 | 0.202 | 3.464 |
| Cytotoxic T Lymphocyte-mediated Apoptosis of Target Cells | 2.94 | 0.294 | 2.449 |
| Regulation Of The Epithelial Mesenchymal Transition In Development Pathway | 2.87 | 0.202 | 3.742 |
| TWEAK Signaling | 2.84 | 0.286 | 1.897 |
| Integrin Signaling | 2.84 | 0.15 | 4.914 |
| Tumoricidal Function of Hepatic Natural Killer Cells | 2.73 | 0.333 | 2.646 |
| Ephrin Receptor Signaling | 2.72 | 0.153 | 3.5 |
| NER Pathway | 2.71 | 0.184 | 2.828 |
| Role of Pattern Recognition Receptors in Recognition of Bacteria and Viruses | 2.71 | 0.162 | 3.742 |
| Wnt/β-catenin Signaling | 2.67 | 0.156 | 1.4 |
| Complement System | 2.67 | 0.27 | 1.414 |
| Protein Kinase A Signaling | 2.52 | 0.125 | 0.686 |
| fMLP Signaling in Neutrophils | 2.5 | 0.172 | 3.207 |
| LPS/IL-1 Mediated Inhibition of RXR Function | 2.47 | 0.142 | 0.943 |
| Actin Cytoskeleton Signaling | 2.41 | 0.141 | 4.2 |
| GM-CSF Signaling | 2.36 | 0.2 | 2.496 |
| VDR/RXR Activation | 2.36 | 0.192 | 0.707 |
| HGF Signaling | 2.34 | 0.167 | 3.357 |
| HMGB1 Signaling | 2.32 | 0.152 | 4.583 |
| Small Cell Lung Cancer Signaling | 2.32 | 0.197 | 2.333 |
| Th1 Pathway | 2.31 | 0.165 | 2.84 |
| Semaphorin Neuronal Repulsive Signaling Pathway | 2.29 | 0.158 | 0.853 |
| PI3K/AKT Signaling | 2.28 | 0.146 | 2.673 |
| Acute Myeloid Leukemia Signaling | 2.23 | 0.18 | 2.53 |
| TNFR2 Signaling | 2.13 | 0.267 | 1.89 |
| Role of NFAT in Regulation of the Immune Response | 2.12 | 0.144 | 4.472 |
| mTOR Signaling | 2.1 | 0.138 | 3.464 |
| Glutathione Redox Reactions I | 2.1 | 0.292 | 2.646 |
| Androgen Signaling | 2.09 | 0.154 | 1.667 |
| Cardiac Hypertrophy Signaling (Enhanced) | 2.09 | 0.115 | 6.934 |
| Toll-like Receptor Signaling | 2.07 | 0.184 | 2.496 |
| iNOS Signaling | 2.07 | 0.222 | 2.828 |
| Mouse Embryonic Stem Cell Pluripotency | 2 | 0.165 | 3.638 |

| **Ingenuity Canonical Pathways** | **-log(BH p-value)** | **Ratio** | **z-score** |
| --- | --- | --- | --- |
| 1. **Pathways from down-regulated DEGs** | | | |
| Synaptogenesis Signaling Pathway | 24.5 | 0.288 | -8.013 |
| Endocannabinoid Neuronal Synapse Pathway | 16 | 0.359 | -4.727 |
| Role of NFAT in Cardiac Hypertrophy | 14.1 | 0.271 | -6.456 |
| Opioid Signaling Pathway | 13 | 0.247 | -6.04 |
| GNRH Signaling | 10.2 | 0.26 | -5.477 |
| Calcium Signaling | 10.2 | 0.243 | -6.091 |
| G Beta Gamma Signaling | 9.03 | 0.287 | -5.396 |
| Dopamine-DARPP32 Feedback in cAMP Signaling | 8.81 | 0.252 | -4.902 |
| Synaptic Long Term Depression | 8.38 | 0.233 | -5.555 |
| GPCR-Mediated Nutrient Sensing in Enteroendocrine Cells | 8.26 | 0.286 | -5.303 |
| Neuropathic Pain Signaling In Dorsal Horn Neurons | 8.22 | 0.297 | -5.477 |
| Reelin Signaling in Neurons | 7.97 | 0.27 | -5.657 |
| Netrin Signaling | 7.85 | 0.354 | -4.796 |
| Synaptic Long Term Potentiation | 7.38 | 0.256 | -5.209 |
| CCR5 Signaling in Macrophages | 7.09 | 0.287 | -3.464 |
| Melatonin Signaling | 6.93 | 0.319 | -3.545 |
| CXCR4 Signaling | 6.58 | 0.222 | -4.041 |
| Protein Kinase A Signaling | 6.55 | 0.165 | -2.84 |
| Signaling by Rho Family GTPases | 6.44 | 0.19 | -5.24 |
| Corticotropin Releasing Hormone Signaling | 6.17 | 0.228 | -1.095 |
| Cardiac Hypertrophy Signaling | 5.91 | 0.188 | -5.754 |
| Apelin Endothelial Signaling Pathway | 5.85 | 0.243 | -3.657 |
| Sperm Motility | 5.57 | 0.188 | -5.099 |
| Thrombin Signaling | 5.54 | 0.192 | -4.271 |
| Nitric Oxide Signaling in the Cardiovascular System | 5.54 | 0.253 | -4.491 |
| Cardiac β-adrenergic Signaling | 5.53 | 0.22 | -2.837 |
| Androgen Signaling | 5.38 | 0.221 | -4.583 |
| α-Adrenergic Signaling | 5.25 | 0.25 | -3.873 |
| Superpathway of Inositol Phosphate Compounds | 5.24 | 0.191 | -6.083 |
| Type II Diabetes Mellitus Signaling | 4.99 | 0.211 | -3.051 |
| Gαq Signaling | 4.94 | 0.203 | -5.196 |
| Glutamate Receptor Signaling | 4.75 | 0.298 | -1.89 |
| cAMP-mediated signaling | 4.6 | 0.175 | -5.096 |
| Huntington's Disease Signaling | 4.58 | 0.173 | -1.706 |
| nNOS Signaling in Neurons | 4.57 | 0.319 | -3 |
| 3-phosphoinositide Biosynthesis | 4.5 | 0.193 | -5.568 |
| White Adipose Tissue Browning Pathway | 4.44 | 0.209 | -4.426 |
| CREB Signaling in Neurons | 4.42 | 0.134 | -7.778 |
| Insulin Secretion Signaling Pathway | 4.3 | 0.168 | -5.778 |
| FcγRIIB Signaling in B Lymphocytes | 4.27 | 0.253 | -2.449 |
| Renin-Angiotensin Signaling | 4.22 | 0.212 | -4.583 |
| Adrenomedullin signaling pathway | 4.18 | 0.178 | -5.657 |
| D-myo-inositol-5-phosphate Metabolism | 4.18 | 0.191 | -5.385 |
| 14-3-3-mediated Signaling | 4.15 | 0.205 | -3.838 |
| Endothelin-1 Signaling | 3.84 | 0.176 | -4.131 |
| Phospholipase C Signaling | 3.8 | 0.158 | -4.914 |
| Relaxin Signaling | 3.74 | 0.187 | -3.606 |
| D-myo-inositol (1,4,5,6)-Tetrakisphosphate Biosynthesis | 3.74 | 0.19 | -5.099 |
| D-myo-inositol (3,4,5,6)-tetrakisphosphate Biosynthesis | 3.74 | 0.19 | -5.099 |
| Cholecystokinin/Gastrin-mediated Signaling | 3.74 | 0.202 | -4.583 |
| P2Y Purigenic Receptor Signaling Pathway | 3.72 | 0.197 | -4.264 |
| Apelin Cardiomyocyte Signaling Pathway | 3.58 | 0.212 | -4.583 |
| CDK5 Signaling | 3.49 | 0.204 | -2.4 |
| CCR3 Signaling in Eosinophils | 3.46 | 0.194 | -3.742 |
| 3-phosphoinositide Degradation | 3.46 | 0.179 | -5.196 |
| Chemokine Signaling | 3.41 | 0.225 | -3.638 |
| Aldosterone Signaling in Epithelial Cells | 3.38 | 0.177 | -4.359 |
| Cardiac Hypertrophy Signaling (Enhanced) | 3.13 | 0.129 | -7.366 |
| PKCθ Signaling in T Lymphocytes | 3.13 | 0.174 | -3.742 |
| Endocannabinoid Developing Neuron Pathway | 3.11 | 0.191 | -1.606 |
| Sphingosine-1-phosphate Signaling | 3.01 | 0.188 | -2.828 |
| ErbB Signaling | 2.99 | 0.202 | -4.243 |
| UVB-Induced MAPK Signaling | 2.9 | 0.25 | -3.606 |
| p70S6K Signaling | 2.82 | 0.178 | -3.13 |
| Rac Signaling | 2.82 | 0.182 | -4.243 |
| Tec Kinase Signaling | 2.75 | 0.162 | -4.123 |
| RhoA Signaling | 2.73 | 0.179 | -3.13 |
| Pregnenolone Biosynthesis | 2.73 | 0.462 | -2.449 |
| Gαs Signaling | 2.73 | 0.187 | -3.3 |
| IL-1 Signaling | 2.69 | 0.196 | -1.89 |
| fMLP Signaling in Neutrophils | 2.69 | 0.181 | -4.243 |
| Ephrin B Signaling | 2.52 | 0.208 | -3.317 |
| HGF Signaling | 2.51 | 0.175 | -4.243 |
| RhoGDI Signaling | 2.51 | 0.153 | 2.828 |
| Ephrin Receptor Signaling | 2.51 | 0.153 | -4.583 |
| GPCR-Mediated Integration of Enteroendocrine Signaling Exemplified by an L Cell | 2.47 | 0.205 | -0.258 |
| IL-8 Signaling | 2.44 | 0.15 | -4.796 |
| Leptin Signaling in Obesity | 2.41 | 0.203 | -2.236 |
| Histidine Degradation VI | 2.4 | 0.4 | -2.449 |
| B Cell Receptor Signaling | 2.31 | 0.151 | -4.041 |
| Role of NFAT in Regulation of the Immune Response | 2.17 | 0.149 | -3.838 |
| PI3K Signaling in B Lymphocytes | 2.11 | 0.159 | -4.472 |
| Ubiquinol-10 Biosynthesis (Eukaryotic) | 2.09 | 0.353 | -2.449 |
| ERK/MAPK Signaling | 2.09 | 0.144 | -3 |
| Amyotrophic Lateral Sclerosis Signaling | 2.05 | 0.175 | -2.111 |
| RANK Signaling in Osteoclasts | 2.04 | 0.18 | -4 |
| Neuroinflammation Signaling Pathway | 2.03 | 0.13 | -2.785 |
| UVA-Induced MAPK Signaling | 2.01 | 0.173 | -3.464 |

**Supplementary Table 8.** Ingenuity Pathway Analysis (IPA) upstream regulator analysis results for **a.** up and **b.** down-regulated differentially expressed genes (DEGs) present in all three glioblastoma (GBM) datasets and identified by the fused inverse-normal (FIN) method.

| Upstream Regulator | mean\|logFC\| | Molecule Type | Activation z-score | P-value overlap | BH p-value |
| --- | --- | --- | --- | --- | --- |
| 1. Upstream regulators for up-regulated DEGs | | | | | |
| TGFB1 | 1.041 | growth factor | 8.971 | 2.51E-88 | 2.08E-84 |
| TP53 | 2.208 | transcription regulator | 3.96 | 9.95E-80 | 4.14E-76 |
| ERBB2 | 1.26 | kinase | 7.269 | 2.60E-66 | 3.60E-63 |
| MYC | 2.687 | transcription regulator | 4.855 | 4.37E-62 | 4.55E-59 |
| CDKN1A | 2.457 | kinase | -4.091 | 2.34E-56 | 1.77E-53 |
| NFKBIA | 1.272 | transcription regulator | 3.344 | 1.80E-43 | 7.47E-41 |
| FOXM1 | 4.83 | transcription regulator | 7.238 | 5.80E-39 | 1.79E-36 |
| EGFR | 4.605 | kinase | 5.001 | 9.51E-37 | 2.64E-34 |
| CCND1 | 1.251 | transcription regulator | 4.578 | 2.88E-33 | 5.44E-31 |
| YAP1 | 1.162 | transcription regulator | 5.297 | 7.46E-32 | 1.29E-29 |
| TWIST1 | 1.889 | transcription regulator | 4.192 | 7.09E-30 | 1.02E-27 |
| E2F1 | 1.146 | transcription regulator | 5.853 | 1.04E-28 | 1.35E-26 |
| TBX2 | 3.103 | transcription regulator | 4.433 | 7.35E-28 | 8.86E-26 |
| NUPR1 | 1.918 | transcription regulator | -3.927 | 1.43E-26 | 1.51E-24 |
| AR | 2.049 | ligand-dependent nuclear receptor | 3.669 | 1.19E-25 | 1.15E-23 |
| JUN | 1.495 | transcription regulator | 5.72 | 2.87E-25 | 2.57E-23 |
| NR1H3 | 1.074 | ligand-dependent nuclear receptor | 2.203 | 1.23E-24 | 1.05E-22 |
| AKT1 | 1.066 | kinase | 4.256 | 3.66E-24 | 2.92E-22 |
| VEGFA | 3.776 | growth factor | 6.513 | 1.21E-23 | 9.28E-22 |
| SPI1 | 1.254 | transcription regulator | 1.876 | 1.63E-23 | 1.22E-21 |
| TCF3 | 1.887 | transcription regulator | -0.806 | 9.61E-23 | 6.50E-21 |
| FAS | 1.754 | transmembrane receptor | -2.084 | 2.13E-22 | 1.42E-20 |
| TGFB2 | 1.198 | growth factor | 3.424 | 5.11E-22 | 3.32E-20 |
| CD44 | 2.568 | other | 4.541 | 2.28E-20 | 1.27E-18 |
| APOE | 1.358 | transporter | -4.791 | 2.73E-19 | 1.39E-17 |
| IRF1 | 2.009 | transcription regulator | 5.486 | 9.97E-19 | 4.74E-17 |
| CEBPB | 1.262 | transcription regulator | 5.281 | 1.01E-18 | 4.77E-17 |
| FN1 | 2.971 | enzyme | 5.104 | 2.56E-17 | 1.10E-15 |
| CDK4 | 3.341 | kinase | 0.469 | 6.10E-17 | 2.51E-15 |
| ANGPT2 | 3.221 | growth factor | 6.136 | 6.54E-17 | 2.68E-15 |
| CCN2 | 1.487 | growth factor | 4.292 | 5.92E-16 | 2.16E-14 |
| E2F2 | 5.191 | transcription regulator | 2.494 | 8.16E-16 | 2.95E-14 |
| EZH2 | 3.461 | transcription regulator | 0.551 | 8.72E-16 | 3.14E-14 |
| NOTCH3 | 2.12 | transcription regulator | 4.045 | 1.01E-15 | 3.60E-14 |
| IRF7 | 1.324 | transcription regulator | 6.854 | 1.06E-15 | 3.73E-14 |
| RBL1 | 1.244 | transcription regulator | -4.344 | 4.95E-15 | 1.62E-13 |
| F2R | 3.98 | G-protein coupled receptor | 5.338 | 6.13E-15 | 2.00E-13 |
| BIRC5 | 4.835 | other | -1.709 | 1.25E-14 | 3.99E-13 |
| S100A9 | 3.908 | other | 3.047 | 1.42E-14 | 4.51E-13 |
| CD40 | 1.101 | transmembrane receptor | 4.744 | 1.43E-14 | 4.51E-13 |
| NAMPT | 2.502 | cytokine | 2.682 | 1.61E-14 | 5.07E-13 |
| MYBL2 | 6.787 | transcription regulator | 2.79 | 2.89E-14 | 8.80E-13 |
| ABCB4 | 2.036 | transporter | -4.078 | 4.01E-14 | 1.18E-12 |
| JAG1 | 2.726 | growth factor | 4.426 | 7.45E-14 | 2.14E-12 |
| COL18A1 | 1.65 | other | -4.828 | 7.84E-14 | 2.22E-12 |
| PLK1 | 2.524 | kinase | -2.02 | 8.98E-14 | 2.51E-12 |
| ACVRL1 | 1.465 | kinase | -0.024 | 1.80E-13 | 4.82E-12 |
| S100A6 | 2.611 | transporter | 0.943 | 2.05E-13 | 5.45E-12 |
| CIP2A | 1.493 | other | -1.763 | 2.76E-13 | 7.26E-12 |
| HOXA10 | 7.693 | transcription regulator | -2.33 | 2.84E-13 | 7.44E-12 |
| RARA | 1.599 | ligand-dependent nuclear receptor | 3.454 | 3.26E-13 | 8.42E-12 |
| TEAD4 | 2.789 | transcription regulator | 4.204 | 3.84E-13 | 9.81E-12 |
| IL33 | 1.493 | cytokine | 5.613 | 4.17E-13 | 1.06E-11 |
| DCN | 1.381 | other | -1.12 | 7.13E-13 | 1.77E-11 |
| S100A8 | 4.872 | other | 2.378 | 7.63E-13 | 1.89E-11 |
| ID3 | 2.405 | transcription regulator | -0.307 | 1.94E-12 | 4.46E-11 |
| BCL6 | 1.086 | transcription regulator | -1.729 | 4.74E-12 | 1.04E-10 |
| CCN1 | 2.124 | other | 3.663 | 7.29E-12 | 1.56E-10 |
| MYD88 | 1.788 | other | 6.715 | 1.37E-11 | 2.83E-10 |
| TREM1 | 4.669 | transmembrane receptor | 3.236 | 2.24E-11 | 4.50E-10 |
| TNFSF13B | 1.681 | cytokine | 3.489 | 2.32E-11 | 4.64E-10 |
| ENG | 1.746 | transmembrane receptor | 0.342 | 2.82E-11 | 5.59E-10 |
| HMOX1 | 2.464 | enzyme | -1.425 | 4.55E-11 | 8.73E-10 |
| TEAD2 | 3.281 | transcription regulator | 4 | 7.47E-11 | 1.40E-09 |
| SPP1 | 1.055 | cytokine | 5.002 | 9.83E-11 | 1.79E-09 |
| IL10RA | 1.235 | transmembrane receptor | -0.431 | 1.01E-10 | 1.84E-09 |
| SMAD1 | 1.086 | transcription regulator | 3.773 | 1.03E-10 | 1.87E-09 |
| CXCR4 | 2.307 | G-protein coupled receptor | 2.791 | 1.18E-10 | 2.12E-09 |
| ETS1 | 1.474 | transcription regulator | 4.918 | 1.43E-10 | 2.55E-09 |
| NOX4 | 2.202 | enzyme | 3.208 | 2.56E-10 | 4.46E-09 |
| MAP3K1 | 1.417 | kinase | 4.376 | 2.69E-10 | 4.66E-09 |
| ATF3 | 1.086 | transcription regulator | -1.834 | 3.38E-10 | 5.79E-09 |
| THBS1 | 2.392 | other | 0.938 | 4.76E-10 | 8.00E-09 |
| WWTR1 | 2.559 | transcription regulator | 2.275 | 5.97E-10 | 9.83E-09 |
| GLIS2 | 1.085 | transcription regulator | -3.45 | 6.95E-10 | 1.14E-08 |
| RUNX3 | 1.993 | transcription regulator | -3.767 | 8.63E-10 | 1.39E-08 |
| KLF6 | 1.287 | transcription regulator | 1.665 | 9.32E-10 | 1.49E-08 |
| TFAP2A | 3.639 | transcription regulator | -1.105 | 9.69E-10 | 1.54E-08 |
| PLAU | 4.022 | peptidase | 2.262 | 1.38E-09 | 2.14E-08 |
| CHEK1 | 2.576 | kinase | 1.233 | 1.75E-09 | 2.67E-08 |
| PTGER4 | 1.554 | G-protein coupled receptor | -1.729 | 2.73E-09 | 4.06E-08 |
| ETV4 | 3.275 | transcription regulator | 3.774 | 3.55E-09 | 5.16E-08 |
| S100A4 | 3.034 | other | 2.341 | 4.52E-09 | 6.43E-08 |
| MMP9 | 6.815 | peptidase | 3.108 | 5.04E-09 | 7.08E-08 |
| TLR3 | 1.497 | transmembrane receptor | 5.937 | 5.38E-09 | 7.53E-08 |
| TEAD3 | 2.377 | transcription regulator | 3.771 | 6.51E-09 | 9.03E-08 |
| SOX2 | 1.572 | transcription regulator | 0.781 | 7.82E-09 | 1.07E-07 |
| TNFRSF1A | 1.995 | transmembrane receptor | 1.079 | 1.15E-08 | 1.52E-07 |
| ANXA2 | 3.997 | other | -1.574 | 1.25E-08 | 1.64E-07 |
| SNAI2 | 2.56 | transcription regulator | 2.345 | 1.34E-08 | 1.75E-07 |
| HMGA1 | 1.256 | transcription regulator | 1.163 | 1.44E-08 | 1.86E-07 |
| ITGA5 | 2.844 | transmembrane receptor | 2.102 | 1.47E-08 | 1.90E-07 |
| PDGFC | 1.867 | growth factor | 3.388 | 1.79E-08 | 2.30E-07 |
| TYROBP | 1.505 | transmembrane receptor | 2.2 | 2.24E-08 | 2.83E-07 |
| DLL4 | 1.581 | other | -0.357 | 2.27E-08 | 2.86E-07 |
| PRDM1 | 1.411 | transcription regulator | -1.626 | 2.51E-08 | 3.15E-07 |
| SPARC | 2.132 | other | -0.419 | 2.68E-08 | 3.36E-07 |
| ZEB1 | 1.431 | transcription regulator | 1.684 | 4.32E-08 | 5.24E-07 |
| NCF1 | 1.26 | enzyme | 2.905 | 5.57E-08 | 6.62E-07 |
| IGFBP2 | 4.882 | other | 2.823 | 6.06E-08 | 7.13E-07 |
| SOX4 | 2.495 | transcription regulator | 3.934 | 6.18E-08 | 7.26E-07 |
| TIMP1 | 3.632 | cytokine | 1.604 | 1.11E-07 | 1.24E-06 |
| E2F5 | 1.566 | transcription regulator | 2 | 1.15E-07 | 1.28E-06 |
| TLR2 | 1.287 | transmembrane receptor | 1.861 | 1.60E-07 | 1.74E-06 |
| CXCL8 | 3.465 | cytokine | 2.976 | 2.01E-07 | 2.15E-06 |
| ASCL1 | 1.684 | transcription regulator | 2.383 | 2.03E-07 | 2.16E-06 |
| PARP9 | 1.68 | enzyme | 2.373 | 2.29E-07 | 2.42E-06 |
| NEDD9 | 1.814 | other | 1.973 | 2.29E-07 | 2.42E-06 |
| ZMYND10 | 1.329 | other | -0.342 | 2.45E-07 | 2.55E-06 |
| ACKR3 | 1.907 | G-protein coupled receptor | 2.027 | 2.45E-07 | 2.55E-06 |
| MSTN | 1.995 | growth factor | 0.366 | 2.90E-07 | 2.98E-06 |
| SPHK1 | 1.69 | kinase | 1.646 | 3.16E-07 | 3.22E-06 |
| GMNN | 1.505 | transcription regulator | -3.748 | 3.20E-07 | 3.26E-06 |
| WNT5A | 1.846 | cytokine | 1.597 | 3.22E-07 | 3.27E-06 |
| CTSB | 1.196 | peptidase | 2.408 | 3.31E-07 | 3.35E-06 |
| LGALS3 | 3.025 | other | 0.747 | 3.35E-07 | 3.39E-06 |
| BAX | 1.466 | transporter | 2.256 | 3.61E-07 | 3.62E-06 |
| POSTN | 6.219 | other | 2.589 | 3.61E-07 | 3.62E-06 |
| DDB2 | 1.301 | other | 0.354 | 5.65E-07 | 5.46E-06 |
| IFIH1 | 1.048 | enzyme | 3.142 | 6.04E-07 | 5.77E-06 |
| CDK2 | 2.938 | kinase | 2.496 | 7.22E-07 | 6.82E-06 |
| ACTL6A | 1.54 | other | 0.905 | 8.51E-07 | 7.96E-06 |
| MAP3K14 | 1.231 | kinase | 2.881 | 8.79E-07 | 8.19E-06 |
| NFKB2 | 1.33 | transcription regulator | 3.218 | 1.09E-06 | 1.01E-05 |
| RUNX1 | 1.627 | transcription regulator | 1.428 | 1.10E-06 | 1.01E-05 |
| OSMR | 2.989 | transmembrane receptor | 1.4 | 1.13E-06 | 1.04E-05 |
| CASP3 | 1.189 | peptidase | 1.249 | 1.32E-06 | 1.20E-05 |
| GDF15 | 5.161 | growth factor | 0.765 | 1.48E-06 | 1.33E-05 |
| PGF | 2.975 | growth factor | 2.245 | 1.66E-06 | 1.46E-05 |
| PLAUR | 2.196 | transmembrane receptor | 2.61 | 1.66E-06 | 1.46E-05 |
| MUC1 | 2.76 | other | 2.782 | 1.87E-06 | 1.63E-05 |
| BCL6B | 1.668 | transcription regulator | 1.309 | 1.87E-06 | 1.63E-05 |
| ADAMTS12 | 2.3 | peptidase | -3.138 | 1.88E-06 | 1.63E-05 |
| PTGS1 | 2.051 | enzyme | 1.5 | 2.30E-06 | 1.97E-05 |
| TRAF3IP2 | 1.532 | other | 3.939 | 2.71E-06 | 2.29E-05 |
| HSPB1 | 1.863 | other | 0.921 | 2.95E-06 | 2.47E-05 |
| PDGFRA | 2.422 | kinase | 2.21 | 3.02E-06 | 2.51E-05 |
| TYMS | 1.54 | enzyme | -2.213 | 3.10E-06 | 2.55E-05 |
| CEBPD | 2.348 | transcription regulator | 0.231 | 3.11E-06 | 2.56E-05 |
| ICAM1 | 2.224 | transmembrane receptor | 3.418 | 3.40E-06 | 2.79E-05 |
| MEX3A | 3.168 | other | 1.387 | 3.80E-06 | 3.09E-05 |
| MMP2 | 3.159 | peptidase | 2.053 | 3.80E-06 | 3.09E-05 |
| ADAM12 | 2.471 | peptidase | -0.269 | 4.19E-06 | 3.40E-05 |
| ANXA1 | 3.445 | enzyme | -1.1 | 4.31E-06 | 3.48E-05 |
| HLX | 1.738 | transcription regulator | -1.66 | 4.31E-06 | 3.48E-05 |
| NT5E | 1.606 | phosphatase | -1.134 | 5.34E-06 | 4.24E-05 |
| GPX1 | 1.407 | enzyme | -3.532 | 5.80E-06 | 4.59E-05 |
| IGFBP7 | 2.448 | transporter | -0.174 | 5.91E-06 | 4.64E-05 |
| EDNRA | 2.101 | transmembrane receptor | 2.828 | 5.91E-06 | 4.64E-05 |
| ETV1 | 2.044 | transcription regulator | 2.158 | 6.60E-06 | 5.13E-05 |
| TGFB1I1 | 3.679 | transcription regulator | 0.452 | 6.60E-06 | 5.13E-05 |
| SAMSN1 | 1.112 | other | 4.583 | 6.61E-06 | 5.13E-05 |
| CIITA | 1.126 | transcription regulator | 1.576 | 7.27E-06 | 5.59E-05 |
| LATS2 | 1.081 | kinase | 0.971 | 7.27E-06 | 5.59E-05 |
| PIM1 | 1.032 | kinase | 2.179 | 7.28E-06 | 5.60E-05 |
| AURKB | 5.485 | kinase | -1.177 | 9.02E-06 | 6.78E-05 |
| IFI16 | 1.477 | transcription regulator | 3.262 | 9.50E-06 | 7.12E-05 |
| KDR | 1.413 | kinase | 1.105 | 1.02E-05 | 7.60E-05 |
| SHC1 | 1.751 | other | -0.967 | 1.60E-05 | 0.000115 |
| PSMB9 | 1.808 | peptidase | 0.391 | 1.67E-05 | 0.000118 |
| SERPINH1 | 3.581 | other | 2.401 | 1.67E-05 | 0.000118 |
| CPXM1 | 4.813 | peptidase | 2.236 | 1.74E-05 | 0.000121 |
| LEF1 | 1.707 | transcription regulator | 1.929 | 1.90E-05 | 0.000132 |
| ZNF217 | 1.274 | transcription regulator | -1.846 | 2.03E-05 | 0.00014 |
| AIF1 | 1.007 | other | 2.607 | 2.16E-05 | 0.000149 |
| SPRY2 | 1.039 | other | -0.992 | 2.27E-05 | 0.000155 |
| ARHGAP31 | 1.041 | other | -0.905 | 2.81E-05 | 0.000189 |
| SOCS3 | 2.869 | phosphatase | -1.99 | 3.27E-05 | 0.000217 |
| TRIB3 | 2.785 | kinase | -1.897 | 3.29E-05 | 0.000218 |
| TNFAIP3 | 1.782 | enzyme | -2.028 | 3.63E-05 | 0.000237 |
| PLAT | 2.471 | peptidase | 2.921 | 3.86E-05 | 0.000251 |
| AEBP1 | 2.812 | peptidase | -0.403 | 3.89E-05 | 0.000252 |
| CYBA | 1.593 | enzyme | 2.414 | 3.89E-05 | 0.000252 |
| RUVBL1 | 1.297 | transcription regulator | 1.808 | 3.92E-05 | 0.000252 |
| SNHG1 | 1.044 | other | -0.243 | 3.92E-05 | 0.000252 |
| LAMC1 | 2.891 | other | -2.616 | 4.03E-05 | 0.000256 |
| ID4 | 1.659 | transcription regulator | -1.951 | 4.03E-05 | 0.000256 |
| HOXA7 | 7.114 | transcription regulator | 2.547 | 4.67E-05 | 0.000294 |
| C3AR1 | 1.143 | G-protein coupled receptor | 2.107 | 5.03E-05 | 0.000317 |
| EPHA2 | 2.476 | kinase | 1.977 | 5.20E-05 | 0.000326 |
| NGFR | 2.284 | transmembrane receptor | -0.328 | 5.22E-05 | 0.000326 |
| TNFRSF1B | 1.187 | transmembrane receptor | 3.29 | 5.64E-05 | 0.000348 |
| MCM7 | 1.516 | enzyme | NA | 5.68E-05 | 0.000348 |
| CKS1B | 1.363 | kinase | 2.215 | 5.68E-05 | 0.000348 |
| C3 | 1.272 | peptidase | 2.563 | 6.00E-05 | 0.000367 |
| C1QA | 2.385 | other | 1.237 | 6.51E-05 | 0.000395 |
| CRNDE | 2.876 | other | 2.37 | 6.99E-05 | 0.000424 |
| HAS2 | 3.55 | enzyme | 0.808 | 7.98E-05 | 0.000475 |
| CDK1 | 3.762 | kinase | 2.213 | 8.07E-05 | 0.000478 |
| RND3 | 2.011 | enzyme | 1.945 | 8.07E-05 | 0.000478 |
| LTBR | 1.001 | transmembrane receptor | 1.508 | 8.09E-05 | 0.000479 |
| MAPK7 | 1.248 | kinase | 1.631 | 8.83E-05 | 0.00052 |
| CLU | 1.038 | other | 0.913 | 8.92E-05 | 0.000523 |
| HOXD10 | 8.605 | transcription regulator | 1.343 | 9.14E-05 | 0.000534 |
| SOX11 | 3.377 | transcription regulator | 1.646 | 9.89E-05 | 0.000575 |
| INHBB | 1.78 | growth factor | 2.8 | 0.000108 | 0.000628 |
| EFNA2 | 1.755 | kinase | -2.673 | 0.000109 | 0.000628 |
| ID1 | 1.214 | transcription regulator | 2.043 | 0.000109 | 0.00063 |
| MAFB | 1.242 | transcription regulator | 1.323 | 0.000115 | 0.000665 |
| COL1A1 | 4.721 | other | 1.934 | 0.000117 | 0.000667 |
| GAS2L3 | 3.14 | other | -2.63 | 0.000117 | 0.000667 |
| CBX3 | 1.288 | transcription regulator | NA | 0.000121 | 0.000689 |
| PRKD1 | 1.199 | kinase | 3.532 | 0.000126 | 0.000714 |
| SMO | 2.739 | G-protein coupled receptor | NA | 0.000135 | 0.00076 |
| DES | 2.527 | other | -1.98 | 0.000142 | 0.000789 |
| ELN | 2.609 | other | 0.896 | 0.000149 | 0.000822 |
| CTSS | 1.594 | peptidase | 1.026 | 0.000151 | 0.000834 |
| MEOX2 | 4.66 | transcription regulator | -2.474 | 0.000156 | 0.000859 |
| RIPK1 | 1.161 | kinase | 0.632 | 0.00018 | 0.000978 |
| MELK | 5.434 | kinase | 1.896 | 0.000184 | 0.00098 |
| FZD7 | 2.772 | G-protein coupled receptor | 1 | 0.000184 | 0.00098 |
| BRCA2 | 2.764 | transcription regulator | -1.154 | 0.000184 | 0.00098 |
| HELLS | 1.894 | enzyme | 2 | 0.000185 | 0.00098 |
| NEDD4 | 1.549 | enzyme | -1.671 | 0.000185 | 0.00098 |
| PTTG1 | 3.247 | transcription regulator | -0.238 | 0.000215 | 0.00113 |
| STK40 | 1.112 | kinase | 1.589 | 0.000253 | 0.00131 |
| GAS5 | 1.03 | other | -1.604 | 0.000253 | 0.00131 |
| LOX | 4.548 | enzyme | 1.906 | 0.000253 | 0.00131 |
| PDGFRB | 1.307 | kinase | 0.842 | 0.000258 | 0.00133 |
| ITGB2 | 1.448 | transmembrane receptor | 0.971 | 0.000278 | 0.00143 |
| EFNA4 | 1.655 | kinase | -3.317 | 0.00032 | 0.00161 |
| HEY1 | 1.792 | transcription regulator | -0.97 | 0.000354 | 0.00176 |
| GAPDH | 1.067 | enzyme | -2.109 | 0.000355 | 0.00177 |
| DPP4 | 2.169 | peptidase | 0.283 | 0.000399 | 0.00198 |
| SULF2 | 1.281 | enzyme | 1.391 | 0.000415 | 0.00204 |
| ACTB | 1.024 | other | 0.956 | 0.000415 | 0.00204 |
| BIRC3 | 1.523 | enzyme | -0.216 | 0.000484 | 0.00235 |
| USP18 | 1.777 | peptidase | -2.959 | 0.000487 | 0.00235 |
| MECOM | 1.255 | transcription regulator | 0.577 | 0.000487 | 0.00235 |
| CD14 | 2.631 | transmembrane receptor | 3.24 | 0.000497 | 0.00236 |
| TIMP3 | 1.46 | other | -2.354 | 0.000507 | 0.0024 |
| MKI67 | 5.201 | other | 1.216 | 0.000516 | 0.00242 |
| PARP14 | 1.209 | enzyme | 1.387 | 0.000556 | 0.00257 |
| ITGA4 | 2.774 | transmembrane receptor | -0.129 | 0.000556 | 0.00257 |
| ATF5 | 1.443 | transcription regulator | 1.969 | 0.000556 | 0.00257 |
| CISH | 2.232 | other | -1.175 | 0.000598 | 0.00272 |
| CGAS | 1.247 | enzyme | 2.815 | 0.000651 | 0.00294 |
| CDH2 | 1.052 | other | 0.577 | 0.000655 | 0.00294 |
| NRP1 | 2.015 | transmembrane receptor | 0.128 | 0.000655 | 0.00294 |
| ADM | 3.417 | other | -2.099 | 0.000735 | 0.00328 |
| MMP14 | 3.434 | peptidase | -1.195 | 0.000807 | 0.00359 |
| NLRC5 | 1.94 | transcription regulator | 1.926 | 0.000832 | 0.00368 |
| PROCR | 1.694 | other | 0.788 | 0.000862 | 0.0038 |
| BGN | 1.918 | other | 0.239 | 0.000862 | 0.0038 |
| CALR | 1.239 | transcription regulator | 1.525 | 0.000902 | 0.00396 |
| CD36 | 1.975 | transmembrane receptor | 4.202 | 0.000908 | 0.00398 |
| MCL1 | 1.314 | transporter | 0.871 | 0.000954 | 0.00414 |
| H1-2 | 2.235 | other | -0.42 | 0.000978 | 0.00423 |
| DNASE2 | 1.292 | enzyme | -2.784 | 0.00102 | 0.0044 |
| SOX9 | 1.516 | transcription regulator | 2.165 | 0.00103 | 0.00444 |
| CCR1 | 1.03 | G-protein coupled receptor | 0.895 | 0.00112 | 0.00476 |
| TMPO | 1.043 | other | 2 | 0.00113 | 0.00476 |
| LGALS1 | 2.065 | other | 2.275 | 0.00138 | 0.00574 |
| TNFAIP6 | 1.941 | other | 1.915 | 0.00152 | 0.00624 |
| HNRNPAB | 1.296 | enzyme | 2 | 0.00153 | 0.00624 |
| YBX1 | 1.7 | transcription regulator | 1.719 | 0.00169 | 0.00687 |
| EBF1 | 1.316 | transcription regulator | 3.057 | 0.00197 | 0.00772 |
| AURKA | 2.84 | kinase | 1.023 | 0.00198 | 0.00772 |
| IGFBP5 | 2.229 | other | 2.007 | 0.00201 | 0.00778 |
| ACE | 2.002 | peptidase | 0.816 | 0.00201 | 0.00778 |
| HOXA5 | 7.06 | transcription regulator | 2.361 | 0.00201 | 0.00778 |
| HAND2 | 5.899 | transcription regulator | -0.444 | 0.00205 | 0.00795 |
| EFEMP1 | 2.47 | enzyme | -1.982 | 0.00212 | 0.00807 |
| TSPO | 1.285 | transmembrane receptor | -1 | 0.00212 | 0.00807 |
| PLA2G5 | 2.165 | enzyme | 1.98 | 0.00212 | 0.00807 |
| NR5A2 | 4.148 | ligand-dependent nuclear receptor | 0.978 | 0.00246 | 0.00929 |
| SOX6 | 1.243 | transcription regulator | 0.862 | 0.0026 | 0.00976 |

| Upstream Regulator | mean\|logFC\| | Molecule Type | Activation z-score | P-value overlap | BH p-value |
| --- | --- | --- | --- | --- | --- |
| 1. Upstream regulators for down-regulated DEGs | | | | | |
| TCF7L2 | NA | transcription regulator | -9.736 | 9.79E-31 | 4.22E-27 |
| MAPT | -1.398 | other | 0.728 | 2.66E-24 | 5.74E-21 |
| levodopa | NA | chemical - endogenous mammalian | -3.748 | 2.00E-19 | 2.88E-16 |
| HTT | NA | transcription regulator | 1.919 | 7.36E-17 | 6.91E-14 |
| FMR1 | NA | translation regulator | 5.211 | 8.02E-17 | 6.91E-14 |
| SNCA | -4.209 | enzyme | -1.176 | 3.76E-15 | 2.52E-12 |
| REST | NA | transcription regulator | 4.284 | 4.09E-15 | 2.52E-12 |
| BDNF | NA | growth factor | -5.649 | 6.51E-12 | 3.51E-09 |
| DSCAML1 | -2.164 | other | -3.651 | 9.02E-12 | 4.32E-09 |
| HDAC4 | NA | transcription regulator | -0.863 | 1.16E-10 | 4.98E-08 |
| DSCAM | NA | other | -4.163 | 2.22E-10 | 8.70E-08 |
| MKNK1 | NA | kinase | -5.477 | 3.02E-10 | 1.08E-07 |
| topotecan | NA | chemical drug | 4.753 | 4.85E-10 | 1.50E-07 |
| CREB1 | NA | transcription regulator | 0.459 | 4.86E-10 | 1.50E-07 |
| PHF21A | NA | other | -2.51 | 1.43E-09 | 4.12E-07 |
| MECP2 | NA | transcription regulator | -2.411 | 6.96E-09 | 1.76E-06 |
| tetrodotoxin | NA | chemical drug | 2.219 | 1.34E-08 | 3.21E-06 |
| Calmodulin | NA | group | -2.059 | 1.87E-08 | 4.25E-06 |
| PSEN1 | -1.141 | peptidase | -0.722 | 4.16E-08 | 8.96E-06 |
| GRIN3A | NA | ion channel | 5.292 | 8.98E-08 | 1.84E-05 |
| DMD | NA | other | -1.32 | 3.12E-07 | 6.11E-05 |
| APP | -1.079 | other | 0.476 | 1.92E-06 | 0.000359 |
| NFASC | -2.903 | other | -2.952 | 3.81E-06 | 0.000684 |
| tazemetostat | NA | chemical drug | -2.929 | 4.89E-06 | 0.000842 |
| SLC30A3 | NA | transporter | -2.433 | 1.96E-05 | 0.00325 |
| SP2509 | NA | chemical reagent | -2.771 | 2.79E-05 | 0.00444 |
| MFSD2A | NA | transporter | 2.949 | 2.89E-05 | 0.00444 |
| JAK1/2 | NA | group | -4.123 | 4.19E-05 | 0.0061 |
| D-2-amino-5-phosphonovaleric acid | NA | chemical reagent | 0.152 | 4.39E-05 | 0.0061 |
